# Prostate Cancer: Dissecting Novel Immunosuppressive Mechanisms Through Context-Specific Transcriptomic Programs and MDSC Cells

**DOI:** 10.3390/ijms27031511

**Published:** 2026-02-03

**Authors:** Pedro Reyes Martinez, Erick Sierra Diaz, Fabiola Solorzano Ibarra, Jorge Raul Vazquez Urrutia, José de Jesús Guerrero García, Martha Cecilia Téllez Bañuelos, Julio Enrique Castañeda Delgado, Karina Sanchez Reyes, Pablo Cesar Ortiz Lazareno

**Affiliations:** 1 Doctorado en Ciencias Biomédicas, Departamento de Fisiología, Centro Universitario de Ciencias de la Salud (CUCS), Universidad de Guadalajara, Guadalajara 44350, Mexico; pedro.reyesm97@gmail.com; 2 División de Epidemiología, Unidad Médica de Alta Especialidad (UMAE), Hospital de Especialidades (HE), Centro Médico Nacional de Occidente (CMNO), Instituto Mexicano del Seguro Social (IMSS), Guadalajara 44329, Mexico; erksland@gmail.com; 3 División de Inmunología, Centro de Investigación Biomédica de Occidente (CIBO), Centro Médico Nacional de Occidente (CMNO), Instituto Mexicano del Seguro Social (IMSS), Guadalajara 44340, Mexico; 4 Estancias Posdoctorales Secihti, Guadalajara 44340, Mexico; fabiolasolorzanoibarra@gmail.com; 5 Department of Medicine, The Pennsylvania State University College of Medicine, The Pennsylvania State University, Hershey, PA 17033, USA; jvazquezurrutia@pennstatehealth.psu.edu; 6 Banco de Sangre Central, Unidad Médica de Alta Especialidad (UMAE), Hospital de Especialidades (HE), Centro Médico Nacional de Occidente (CMNO), Instituto Mexicano del Seguro Social (IMSS), Guadalajara 44329, Mexico; jg1@gmail.com; 7 Laboratorio de Inmunología Traslacional, Departamento de Biología Celular y Molecular, Centro Universitario de Ciencias Biológicas y Agropecuarias, Universidad de Guadalajara, Zapopan 45200, Mexico; martha.tellez@academicos.udg.mx; 8 Unidad de Investigación Biomédica de Zacatecas, Instituto Mexicano del Seguro Social (IMSS), Zacatecas 98000, Mexico; julioenriquecastaneda@gmail.com; 9 Investigadores por México, Cátedras Secihti, Unidad de Investigación Biomédica de Zacatecas, Instituto Mexicano del Seguro Social (IMSS), Zacatecas 98000, Mexico; 10 Departamento de Clínicas Médicas, Centro Universitario de Ciencias de la Salud, Universidad de Guadalajara, Guadalajara 44350, Mexico; karina.sanchez@academicos.udg.mx

**Keywords:** prostate cancer, myeloid-derived suppressor cells (MDSCs), long non-coding RNAs (lncRNAs), immunosuppression, bulk RNA-seq, tumor microenvironment, flow cytometry

## Abstract

Prostate cancer remains largely refractory to immunotherapy, implying the existence of context-specific immune landscape programs that diverge between circulation and tumor. Here, we integrate bulk RNA sequencing from three cohorts (patient peripheral mononuclear cells, primary prostate tissue, and biochemical-recurrence tumors) with multiparameter flow cytometry, unsupervised UMAP/T-REX (Tracking Responders Expanding) mapping, and de novo discovery of long non-coding RNAs (lncRNAs) to characterize context-specific immunoregulation. Patient PBMCs revealed a coherent *IL-1*/*TNF*/*IL-17* inflammatory architecture with strong chemotactic programs and an unexpected neutrophil-like signal despite density-gradient isolation, consistent with low-density PMN-MDSCs. In contrast, tumors broadly repressed chemokines and innate immune mediators, yet upregulated prostate cancer-associated lncRNAs, indicating local immune quiescence coupled with non-coding regulatory programs. Recurrent tumors acquired epithelial–mesenchymal transition and metabolic remodeling, accompanied by relapse-associated lncRNA signatures, whereas long-term nonrecurrent tumors preserved epithelial and stress-response networks. High-dimensional cytometry confirmed discrete, cancer-enriched myeloid clusters expressing CD47, SIRPα, PD-L1, CD73, and Galectin-9. Network analysis highlighted inflammatory hubs (*CXCL2*, *PTGS2*) in PBMCs and loss of mechanotransduction modules in tumors. Structural modeling uncovered a three-way junction and 3′ triple helix in lncRNA. Collectively, these data suggest that circulating inflammatory rewiring is associated with checkpoint-rich suppressor expansion and tumor immune quiescence, outlining integrated myeloid- and RNA-directed strategies for cancer research.

## 1. Introduction

Prostate cancer is amongst the most prevalent malignancies in men, characterized by substantial molecular and cellular heterogeneity, dysregulated androgen-receptor signaling, and profound alterations in the tumor microenvironment [1,2,3,4,5]. Beyond tumor-intrinsic genetic changes, immune evasion has emerged as a central feature of prostate cancer progression, in which antitumor immune responses are progressively attenuated through a process of immunoediting, ultimately favoring immune tolerance and tumor persistence [6,7,8,9].

Multiple immunosuppressive cell populations contribute to this immune-evasive landscape, including regulatory T and B cells, alternatively polarized macrophages, neutrophils, and myeloid-derived suppressor cells (MDSCs) [10]. MDSCs represent a heterogeneous population of immature myeloid cells with potent immunosuppressive features, capable of suppressing T-cell activation through cytokine secretion, metabolic depletion, reactive oxygen species production, and engagement of immunoregulatory receptors such as CD47, SIRPα, PD-L1, CD73, and Galectin-9 [11,12,13,14,15,16,17,18,19,20]. While MDSCs have been extensively characterized in several malignancies, their presence, phenotypic diversity, and functional relevance in prostate cancer remain incompletely defined [21,22,23,24,25,26].

This knowledge gap is particularly relevant given the complex genomic architecture of prostate cancer, which includes extensive mutational and rearrangement burdens, alterations in the androgen receptor pathway, and diverse gene fusions that may shape tumor–immune interactions [27]. A deeper understanding of how systemic immune remodeling, myeloid suppressor expansion, and immunoregulatory signaling intersect in prostate cancer is therefore needed.

In this study, we performed an integrative analysis combining bulk transcriptomics from peripheral blood mononuclear cells (PBMCs) and tumor tissue with high-dimensional flow cytometry and computational lncRNA discovery to characterize immune-associated regulatory programs in prostate cancer. By focusing on systemic myeloid activation, MDSC-associated immunosuppressive pathways, and context-specific transcriptional architectures, we aimed to elucidate mechanisms underlying immune evasion and identify regulatory features associated with disease progression.

## 2. Results

### 2.1. Divergent Systemic and Tumor-Intrinsic Transcriptional Programs Reveal Inflammation, Immune Suppression, and Relapse-Associated lncRNA Signatures in Prostate Cancer

Differential gene expression analysis was performed across three independent RNA-seq cohorts: (i) PBMCs, including 8 prostate cancer patients and 6 healthy controls; (ii) prostate tissue, consisting of 20 tumor samples and 10 healthy prostate samples; and (iii) the relapse cohort, comprising 47 tumor samples from patients who developed biochemical recurrence within 5 years and 43 from patients without recurrence for more than 10 years.

**Figure 1:**
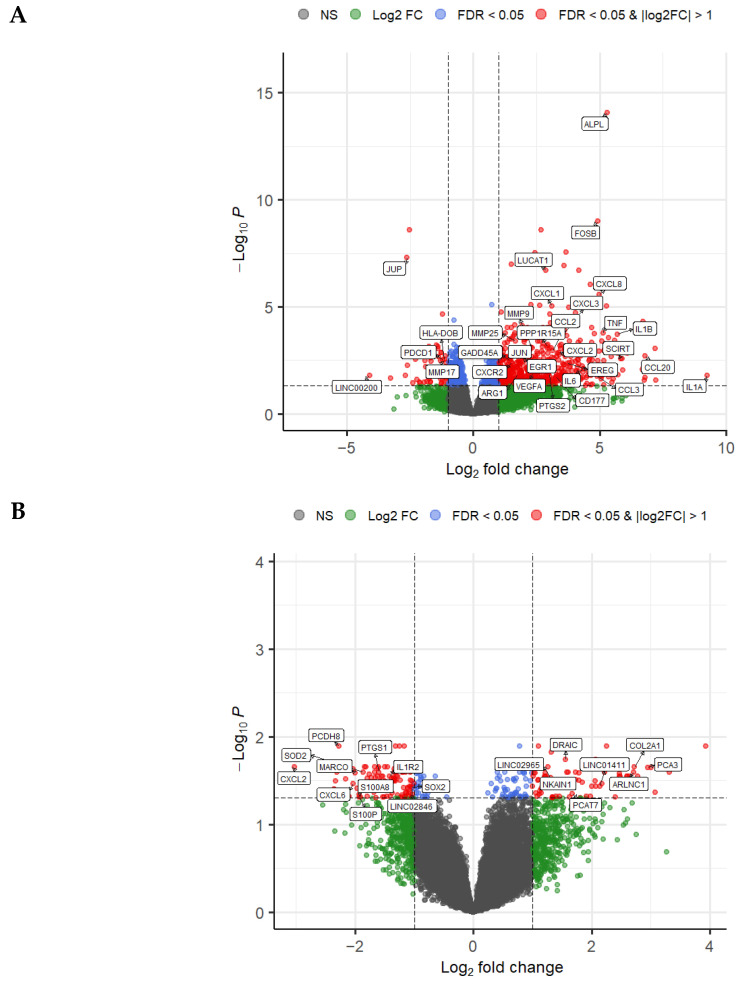

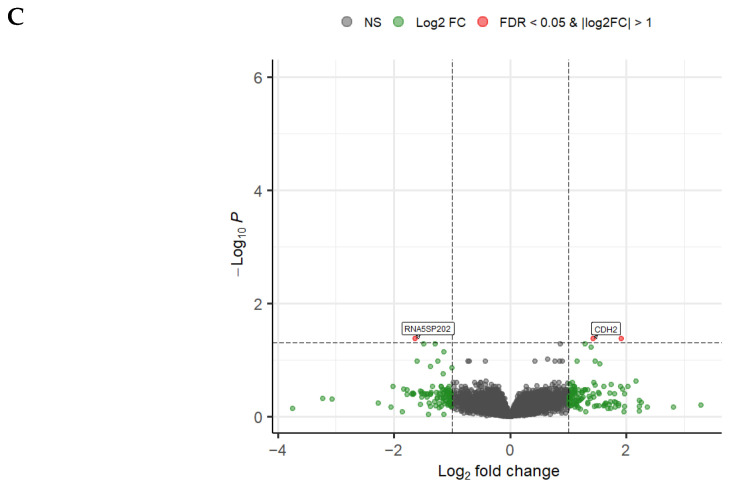
Transcriptomic characterization of prostate cancer across PBMCs and tissue, including relapse-associated expression signatures. (**A**) Volcano plot showing differential gene expression in PBMC RNA-seq data comparing prostate cancer patients (PC, *n* = 8) with healthy controls (HC, n = 6). (**B**) Volcano plot of prostate cancer tissue versus healthy prostate tissue (tumor, n = 20; healthy, n = 10), highlighting tumor-specific transcriptional alterations. (**C**) Volcano plot comparing relapse versus non-relapse prostate cancer tissue (recurrent, n = 47; non-recurrent, n = 43), revealing genes associated with biochemical recurrence. Horizontal dashed lines indicate false discovery rate (FDR < 0.05).

All three cohorts displayed distinct transcriptional alterations (Figure 1A–C). In PBMCs (Figure 1A), the inflammatory signature was highly pronounced, with striking overexpression of pivotal proinflammatory mediators, including *IL1A*, *IL1B*, *IL6*, *TNF*, and *PTGS2*, all of which regulate early innate immune activation. This profile was further amplified by robust upregulation of chemokines fundamental for leukocyte mobilization—*CCL2*, *CCL3*, *CCL20*, *CXCL1*, *CXCL2*, *CXCL3*, and *CXCL8*—highlighting a strong transcriptional program oriented toward neutrophil and monocyte recruitment. Additional inflammation-associated genes, such as *VEGFA*, the stress-response regulator *PPP1R15A*, and the myeloid-linked immunomodulator *ARG1*, were also markedly elevated, collectively indicating a systemic activation of innate immune pathways in circulating immune cells.

Conversely, several genes involved in immune regulation and cellular stress responses—most notably *PDCD1* (programmed cell death 1) and *JUP*—were significantly downregulated, suggesting a loss of inhibitory feedback and a shift toward a hyperinflammatory, dysregulated systemic immune state.

In prostate tumor tissue (Figure 1B), the transcriptional profile revealed a striking and widespread suppression of inflammatory and immune-response genes, consistent with a deeply immunosuppressed tumor microenvironment. In line with this, key chemokines associated with neutrophil and monocyte recruitment, specifically *CXCL2* and *CXCL6*, were significantly downregulated. Other related chemokines, such as *CXCL1*, *CXCL8*, and *CCL2*, followed a similar downward trend but did not reach statistical significance. Likewise, key regulators of innate immunity and epithelial defense—specifically *IL1R2*, *S100A8*, *S100P*, *SOD2*, *MARCO*, and *PTGS1*—showed pronounced downregulation, reflecting a strong attenuation of cytokine signaling, oxidative stress response, and damage-associated molecular pattern (DAMP) sensing. This suppression contrasts sharply with the hyperinflammatory transcriptional program observed in circulating PBMCs, suggesting that prostate tumors actively silence inflammatory axes to diminish local immune surveillance.

In contrast, a distinct group of genes was robustly overexpressed, dominated by transcripts characteristic of prostate cancer biology. Notably, several lncRNAs—including *PCA3*, PCAT7, *ARLNC1*, *LINC02965*, *LINC01411*, and *DRAIC*—were markedly upregulated. The prominence of lncRNAs among the upregulated genes highlights the importance of non-coding regulatory programs in tumor survival, metabolic reprogramming, and immune dampening within the prostate microenvironment.

In the relapse cohort (Figure 1C), differential expression analysis incorporating false discovery rate (FDR) correction revealed a markedly restricted transcriptional signal distinguishing recurrent from non-recurrent tumors. Among all evaluated transcripts, *CDH2* was the only gene to retain statistical significance after multiple-testing correction (FDR < 0.05), making it the most robust and reproducible molecular feature associated with biochemical recurrence. *CDH2* encodes N-cadherin, a canonical mediator of epithelial–mesenchymal transition (EMT) that has been extensively linked to invasive behavior, tumor plasticity, and metastatic competence, thereby providing a biologically coherent anchor for the observed relapse-associated phenotype.

Beyond this stringent significance threshold, a broader set of genes exhibited consistent but nominal differential expression trends that did not persist after FDR correction and were therefore interpreted as exploratory signals. These included transcripts related to lipid metabolism and epithelial differentiation (e.g., *ALOX12B*, *DAPL1*), as well as several lncRNAs (e.g., *FIRRE*, *LINC01187*, *LINC01612*, *LINC02571*), suggesting coordinated but subtle transcriptional shifts that may contribute to relapse biology.

Conversely, tumors from patients who remained recurrence-free for more than 10 years tended to preserve higher expression levels of genes involved in epithelial integrity, redox regulation, and host defense. These included multiple members of the metallothionein family (*MT1G*, *MT1H*, and *MT2P1*) and epithelial or secretory defense genes such as *PIGR*, *CEACAM20*, *RLN1*, *SPINK1*, and *ATOH1*. Although these differences did not meet FDR-adjusted significance criteria, their coherent functional grouping suggests a transcriptional landscape in non-recurrent tumors that may favor epithelial stability and immune surveillance.

### 2.2. Systemic Inflammatory Rewiring in PBMCs Reveals IL-1/TNF/IL-17-Driven Immune Activation in Prostate Cancer

**Figure 2:**
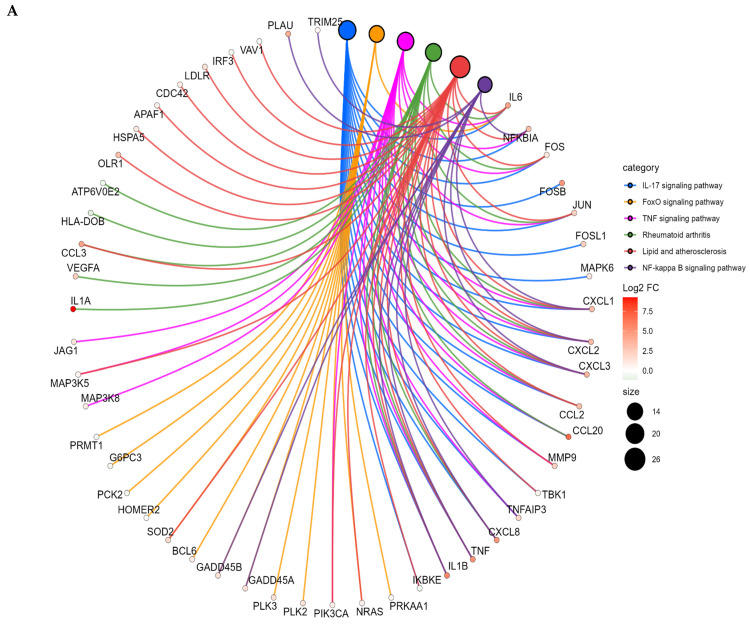

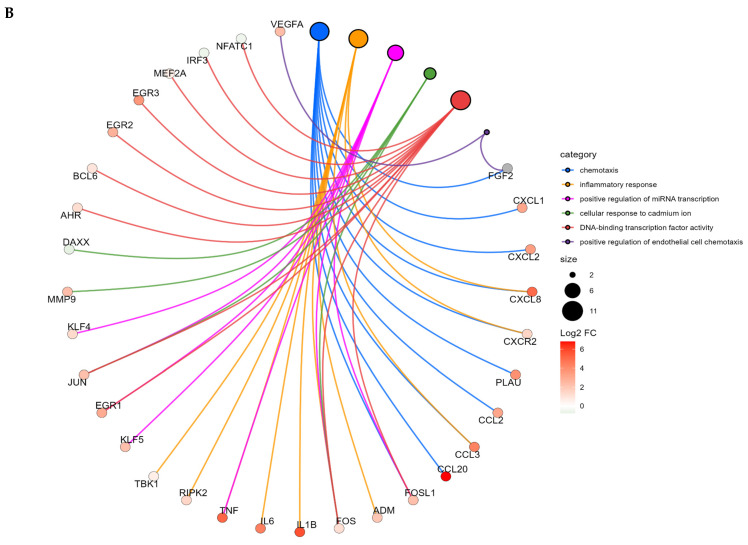
Pathway enrichment network analysis in PBMCs from prostate cancer patients. Circular gene–pathway enrichment networks based on KEGG (**A**) and Gene Ontology (GO) (**B**) analyses of differentially expressed genes in PBMCs from prostate cancer patients (PC, n = 8) compared with healthy controls (HC, n = 6). Outer nodes represent genes contributing to the top significantly enriched pathways (inner nodes). Gene node color reflects log_2_ fold change (red, upregulated), and edge color denotes pathway membership.

Functional enrichment analysis was performed using KEGG and Gene Ontology (GO) databases to identify biological pathways associated with the differentially expressed genes. Among the three cohorts analyzed, statistically significant enrichment was observed exclusively in the PBMC cohort, indicating that transcriptional dysregulation in circulation is organized into highly coordinated biological programs (Figure 2).

Genes upregulated in PBMCs were strongly enriched in central inflammatory and immune signaling pathways, including IL-17 signaling, TNF signaling, NF-κB activation, FoxO signaling, rheumatoid arthritis-related pathways, and lipid-atherosclerosis-associated inflammatory cascades. Pathway–gene networks shown in Figure 2A,B illustrate the dense interconnectivity between key inflammatory mediators—such as *IL1A*, *IL1B*, *IL6*, *TNF*, *NFKBIA*, *CCL2*, *CCL3*, *CCL20*, *CXCL1*, *CXCL2*, *CXCL3*, and *CXCL8*—and major transcriptional regulators, including *JUN*, *FOS*, *FOSL1*, *NRAS*, and *PIK3CA*.

Consistent with these findings, GO-based enrichment revealed significant overrepresentation of processes related to chemotaxis, inflammatory response, positive regulation of mRNA transcription, cellular response to cadmium ion, DNA-binding transcription factor activity, and positive regulation of endothelial cell chemotaxis. The recurrent involvement of *VEGFA*, *CCL2*, *CXCL1*/*2*/*3*/*8*, *JUN*, *FOS*, *EGR1*, and *EGR3* across multiple categories suggests a robust, multi-axis inflammatory program underlying the systemic immune response in prostate cancer.

Collectively, these results indicate that PBMCs from prostate cancer patients exhibit a highly activated inflammatory and chemotactic landscape, driven by convergent IL-1/TNF/IL-17-related pathways. This systemic inflammatory signature contrasts with the immunosuppressive profile observed in tumor tissue, reinforcing a model in which prostate cancer promotes peripheral immune activation despite maintaining local immune dampening within the tumor microenvironment.

### 2.3. Systemic Myeloid Activation Revealed by Aberrant Neutrophil Signatures in PBMCs of Prostate Cancer Patients

**Figure 3:**
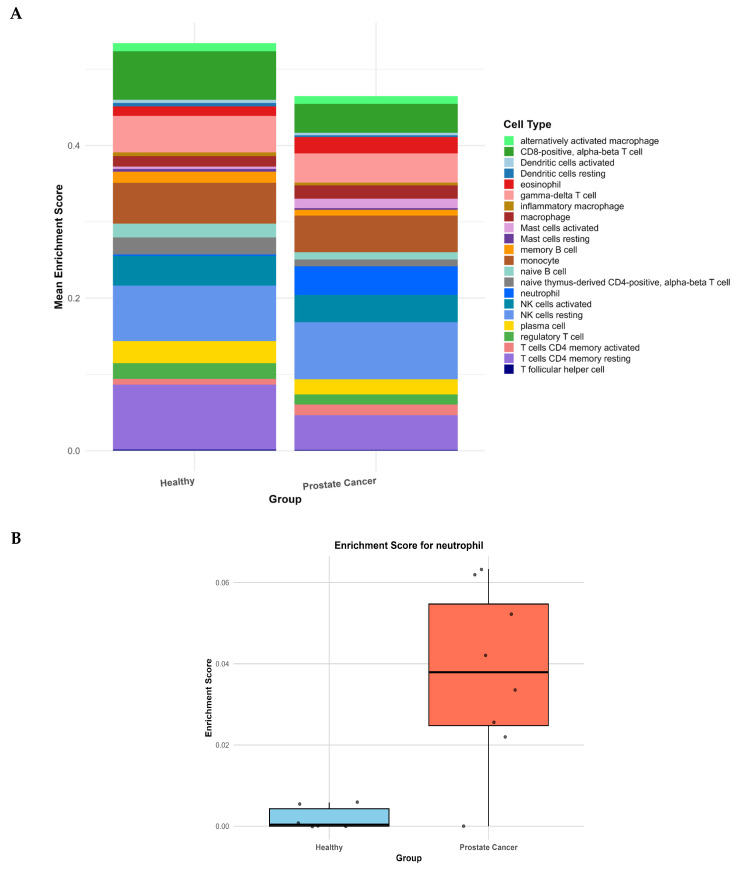
Immune cell enrichment profiles inferred using xCell2. (**A**) Stacked bar plots illustrate relative enrichment scores of immune cell populations in PBMCs from healthy controls (HC, n = 6) and prostate cancer patients (PC, n = 8). (**B**) Boxplot shows that neutrophils are the only immune population significantly increased in prostate cancer patients compared with controls (FDR < 0.05), as assessed by xCell2 deconvolution. Enrichment scores represent relative, unitless xCell2-derived estimates of immune-cell abundance.

Immune cell deconvolution was performed across all three cohorts using xCell2 with the LM22 reference matrix; however, statistically significant differences in immune cell composition were observed only in the PBMC cohort (Figure 3). Across the 22 immune subsets assessed, most populations showed comparable enrichment scores between prostate cancer patients and healthy controls. In contrast, neutrophils showed a marked increase in enrichment score in the cancer group, with clear, statistically significant separation between groups (FDR < 0.05).

This neutrophil enrichment is particularly notable as the analysis was performed exclusively on PBMC-derived RNA, where neutrophil signatures are typically underrepresented due to depletion during density-gradient isolation. The presence of a strong neutrophil-like signature in PBMCs therefore suggests a state of systemic myeloid activation, consistent with the robust upregulation of neutrophil-attracting chemokines (*CXCL1*, *CXCL2*, *CXCL3*, and *CXCL8*) and inflammatory mediators (*IL1A*, *IL1B*, *IL6*, and *TNF*) identified in Figure 1A.

To test whether the neutrophil-like signal detected in PBMCs was driven by a limited set of canonical granulocyte genes, we performed a sensitivity analysis removing 25 well-established neutrophil granule and lineage markers (including *MPO*, *ELANE*, *PRTN3*, *DEFA1*/*3*/*4*, *FCGR3B*, *CEACAM8*, and *S100A8*/*A9*). Reanalysis with xCell2 after gene removal yielded comparable enrichment patterns, and neutrophil scores remained significantly elevated in prostate cancer patients compared with healthy controls (BH-FDR, q = 0.045). These findings indicate that the neutrophil-like signature in PBMCs is not solely attributable to contamination with granulocyte genes (Appendix H, Figure A4).

Collectively, these results demonstrate that prostate cancer is associated with peripheral activation of neutrophil-like myeloid transcriptional programs, reinforcing the concept of a systemic proinflammatory environment that contrasts with the immunosuppressive transcriptional landscape observed within tumor tissue.

### 2.4. Gene Co-Expression Network Analysis Across PBMC, Tumor, and Relapse Cohorts

**Figure 4:**
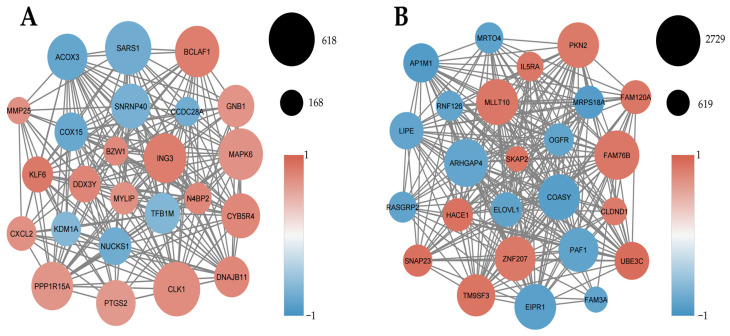
Inflammatory and chemotactic gene co-expression programs in PBMCs from prostate cancer patients. Weighted gene co-expression network analysis (WGCNA) of PBMC RNA-seq data from prostate cancer patients (PC, n = 6) and healthy controls (HC, n = 5). Node size reflects intramodular connectivity, and node color indicates the direction of gene–phenotype correlation (red, positive correlation with prostate cancer; blue, negative correlation). (**A**) The MEblue module shows the strongest positive correlation with prostate cancer (r = 0.66, *p* < 0.05) and is enriched for inflammatory and chemotactic genes. (**B**) The MEturquoise module exhibits the strongest negative correlation with prostate cancer (r = −0.81, *p* < 0.01) and is enriched for metabolic and homeostatic processes.

**Figure 5:**
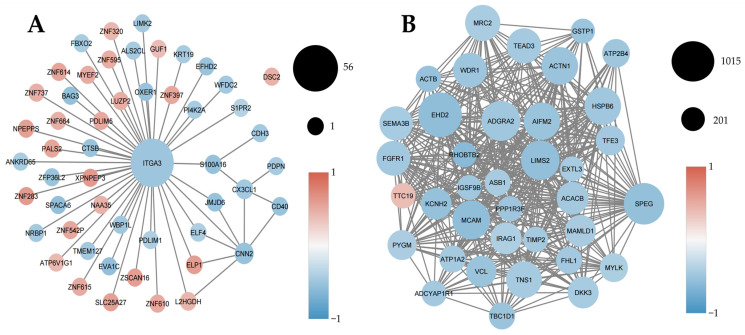
Suppression of epithelial and cytoskeletal co-expression networks in prostate cancer tissue. WGCNA of prostate tumor and non-malignant tissue RNA-seq samples (tumor, n = 19; healthy, n = 10). Node size represents intramodular connectivity, and node color indicates gene–phenotype correlation. (**A**) The MEpurple module displays altered epithelial adhesion and tissue-organization pathways. (**B**) The MEbrown module shows a strong negative correlation with prostate cancer and is enriched for genes involved in cytoskeletal organization and mechanotransduction.

**Figure 6:**
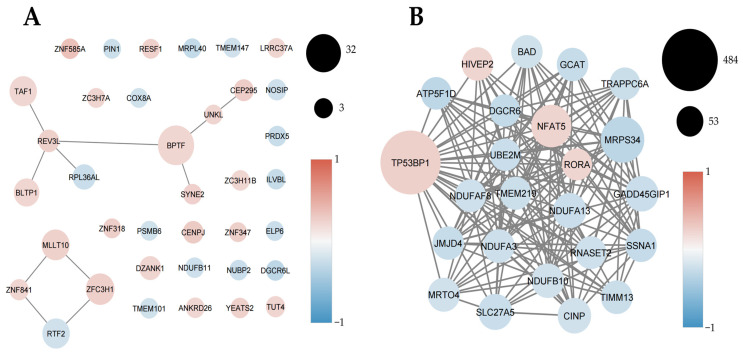
Distinct epigenetic and metabolic co-expression programs associated with biochemical recurrence. WGCNA of the relapse cohort RNA-seq data comparing recurrent (n = 44) and non-recurrent (n = 42) prostate cancer tumors. Node size reflects intramodular connectivity, and node color denotes correlation with recurrence status (red, positive; blue, negative). (**A**) The MEred module is positively associated with biochemical recurrence. (**B**) The MEgreen module is negatively associated with recurrence and enriched for metabolic and stress-response genes.

**Figure A1:**
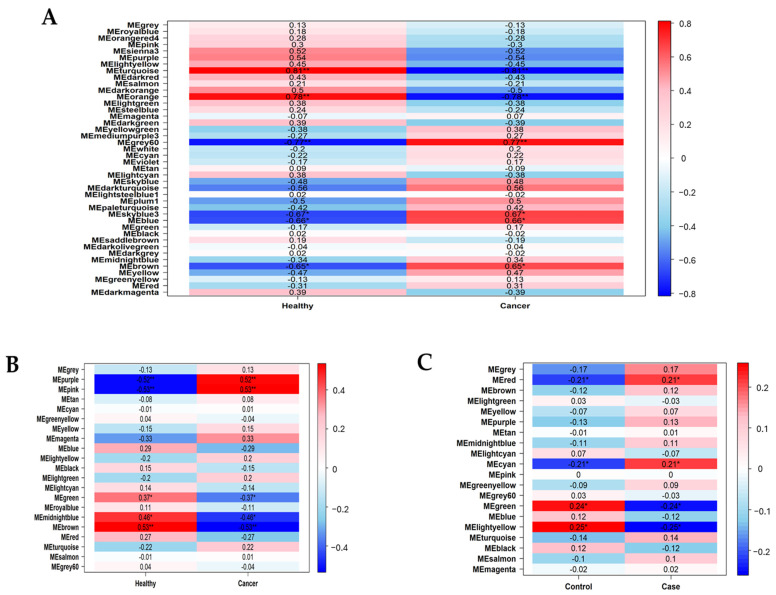
Correlation structure between WGCNA module eigengenes and clinical traits across cohorts. Module-trait heatmaps summarize the Pearson correlation between each module eigengene (ME) and the phenotype of interest. Warmer colors (red) represent positive correlations, whereas cooler colors (blue) reflect negative correlations, and the corresponding *p*-values are displayed within each cell. (**A**) PBMC dataset: identification of modules linked to prostate cancer presence. (**B**) Prostate tissue dataset: detection of modules associated with cancer status. (**C**) Prostate cancer-only dataset: identification of modules related to relapse outcomes. Statistical significance is indicated as follows: * *p* < 0.05 and ** *p* < 0.01.

Weighted gene co-expression network analysis (WGCNA) was performed independently for each cohort to identify coordinated transcriptional programs and the gene communities (modules) underpinning them. Using hierarchical clustering and dynamic tree cutting, genes were grouped into co-expression modules (Appendix A, Figure A1). Network diagrams were then constructed to visualize intra-modular structure, highlight the most interconnected genes (hub genes), and characterize their association with the clinical phenotype (Figure 4, Figure 5 and Figure 6).

#### 2.4.1. Inflammatory and Chemotactic Co-Expression Programs Dominate PBMCs in Prostate Cancer

In the PBMC cohort, co-expression network analysis was performed on 11 high-quality samples (six prostate cancer patients and five healthy controls) retained after quality-control-based outlier removal using hierarchical clustering and principal component analysis (PCA). Weighted gene co-expression network analysis identified the MEblue module as strongly positively associated with prostate cancer (Figure 4A). This module was enriched for inflammatory and chemotactic genes, with key hubs including *CXCL2* and *PTGS2* (COX-2), indicating coordinated activation of immune recruitment and prostaglandin-driven inflammatory signaling in circulating immune cells. These findings are consistent with the differential expression and pathway enrichment analyses (Figure 1 and Figure 2), supporting the presence of a systemic inflammation–dominant transcriptional program in prostate cancer PBMCs.

Conversely, the MEturquoise module showed a strong negative correlation with prostate cancer (Figure 4B) and was enriched for genes involved in metabolic regulation, vesicle trafficking, and cellular homeostasis. Representative hub genes, such as *COASY* and *HACE1*, suggest the suppression of metabolic and cytoprotective programs in circulating immune cells from cancer patients. Together, these modules indicate a shift from homeostatic to inflammatory transcriptional states in PBMCs associated with prostate cancer. Module robustness was further evaluated using a leave-one-out (LOO) jackknife analysis. Only the blue and turquoise modules showed stable eigengene–trait correlations, with complete preservation of correlation sign across all LOO iterations and consistent module composition, as assessed by Jaccard similarity. Other modules displayed unstable correlations and poor compositional preservation and were therefore not considered further (Appendix F and Appendix G; Table A3 and Table A4).

#### 2.4.2. Epithelial and Cytoskeletal Co-Expression Programs Are Suppressed in Prostate Cancer Tissue

In tumor tissue, the MEpurple module displayed a predominantly negative association with prostate cancer. It was centered on genes involved in epithelial adhesion and tissue organization, with *ITGA3* emerging as a key hub (Figure 5A). The coordinated downregulation of epithelial and differentiation-related genes suggests progressive loss of structural integrity during malignant transformation.

Similarly, the MEbrown module showed strong negative correlation with prostate cancer and was enriched for genes regulating cytoskeletal organization and mechanotransduction, including *MYLK*, *VCL*, and *ACTN1* (Figure 5B). The suppression of these modules highlights widespread disruption of epithelial architecture and mechanical stability in prostate cancer tissue.

#### 2.4.3. Epigenetic and Metabolic Co-Expression Programs Distinguish Recurrent from Non-Recurrent Tumors

In the relapse cohort, the MEred module was positively associated with biochemical recurrence and enriched for genes involved in chromatin remodeling and transcriptional regulation, with *BPTF* serving as a central hub (Figure 6A). This module reflects activation of epigenetically driven programs linked to tumor adaptability and recurrence.

Conversely, the MEgreen module showed a strong negative association with recurrence and was enriched for genes involved in metabolic regulation, mitochondrial function, and stress-response pathways, including *NFAT5* and *TP53BP1* (Figure 6B). Preservation of these programs characterized non-recurrent tumors, whereas their loss was associated with relapse.

Together, these findings indicate that biochemical recurrence is associated with the acquisition of epigenetically driven transcriptional programs and the concurrent loss of metabolic and stress-response network integrity.

### 2.5. Discovery and Contextual Characterization of Cohort-Specific lncRNAs in Prostate Cancer

The preceding analyses demonstrated that prostate cancer is characterized by divergent immune-associated transcriptional states, with circulating immune cells exhibiting a coordinated inflammatory and myeloid-skewed program. At the same time, tumor tissue displays broad suppression of inflammatory and chemotactic pathways alongside activation of tumor-intrinsic regulatory networks. Within this framework, lncRNAs emerged as prominent, context-restricted components of these distinct immune states rather than uniformly expressed tumor markers. Building on these cohort-specific transcriptional architectures—defined by systemic inflammation, tumor-intrinsic immune suppression, and relapse-associated regulatory programs—we next sought to assess the contribution of lncRNAs to these divergent biological states. Predictive lncRNA discovery was performed in the PBMC and tumor-tissue cohorts; the relapse cohort was excluded due to insufficient transcriptomic coverage for reliable lncRNA identification. Consistent with this context specificity, lncRNA discovery identified distinct repertoires of non-coding transcripts in PBMCs and tumor tissue. In total, 15 high-confidence lncRNAs were detected in PBMCs and 23 in tumor tissue (>0.99 non-coding probability as estimated by LncDC). These transcripts were subsequently annotated by genomic alignment to GRCh38 using minimap2 and classified according to transcription length, strand orientation, genomic context (antisense, intergenic, sense-intronic, sense-overlapping), and proximity to the nearest protein-coding gene.

Functional characterization of the predicted lncRNAs (Table 1) was performed using LncRTPred and IntaRNA, enabling systematic prediction of putative lncRNA-mRNA interactions across the protein-coding genome. Although LncRTPred yielded numerous high-confidence interactions, only the three most cancer-relevant or immunologically relevant targets per lncRNA were retained for downstream analysis. Targets were prioritized based on predefined biological relevance criteria, including prior annotation in cancer- or immune-associated pathways (KEGG and Gene Ontology), previously reported involvement in prostate cancer progression or myeloid and inflammatory regulation, and functional convergence with the transcriptomic programs enriched in each cohort. IntaRNA was then used to compute minimal hybridization energies for each prioritized pair, generating interaction profiles consistent with stable RNA–RNA contacts and potential post-transcriptional regulatory activity.

**Figure 7:**
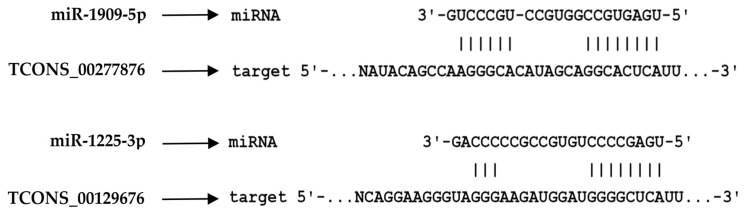
Predicted miRNA–lncRNA interactions supported by seed sequence complementarity and favorable thermodynamic profiles. Only two miRNA–lncRNA interactions met combined statistical and structural criteria, including strong seed complementarity and low hybridization energies. Shown are the interactions between miR-1909-5p and TCONS_00277876 and between miR-1225-3p and TCONS_00129676, highlighting candidate ceRNA-like regulatory axes that may modulate miRNA availability in prostate cancer.

In parallel, miRNA-lncRNA interaction analysis revealed that only two miRNA interactions met statistical and structural criteria, characterized by strong seed complementarity and favorable thermodynamic profiles (Figure 7). These interactions—exemplified by the pairing between miR-1909-5p and TCONS_00277876 and between miR-1225-3p and TCONS_00129676—suggest that a subset of the identified lncRNAs may be compatible with ceRNA-like regulatory axes, potentially modulating miRNA availability in prostate cancer.

### 2.6. Structural Modeling of TCONS_00371831 Reveals Conserved lncRNA Tertiary Motifs

To explore whether any of the newly identified lncRNAs exhibited structural features compatible with known regulatory RNA architectures, we performed de novo tertiary structure modeling using RhoFold+. Because the method has been validated primarily for RNA molecules within a restricted length range, only transcripts of minimal length were technically suitable for reliable modeling.

**Figure 8:**
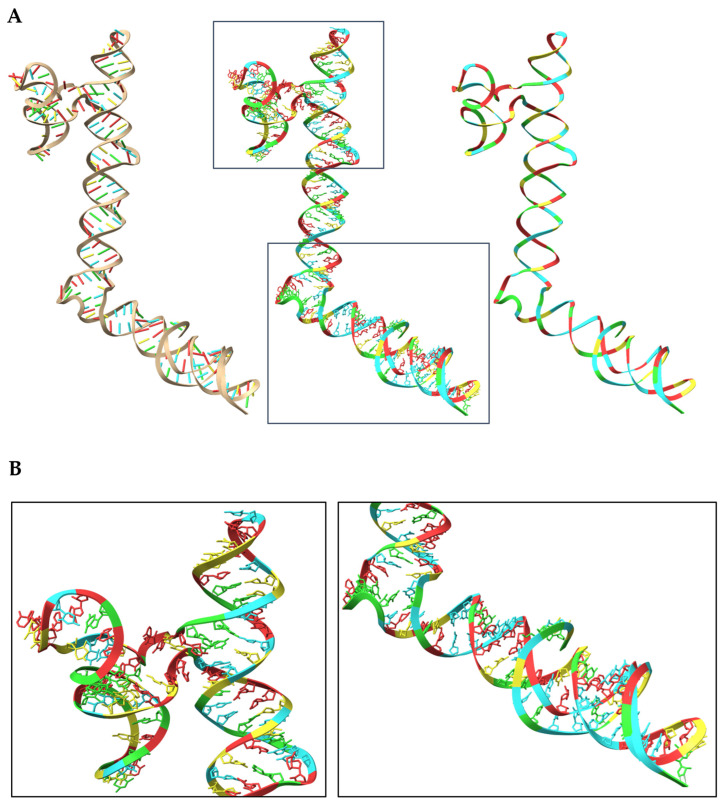
Three-dimensional structural features of the lncRNA TCONS_00371831. (**A**) Predicted three-dimensional model of TCONS_00371831 shown in three complementary visualization styles. The left panel highlights the continuous helical scaffold and long-range coaxial stacking that define global folds of the lncRNA. The central panel, with two zoomed-in insets, reveals internal stem-loop elements, local tertiary contacts, and curved backbone regions that deviate from ideal helicity, illustrating structural heterogeneity along the transcript. The right panel presents a simplified ribbon representation to facilitate visualization of the overall helical trajectory and long-range architectural organization. (**B**) Representative conserved tertiary structural motifs identified within the TCONS_00371831 model. The left panel depicts a parallel Y-shaped three-way junction (3WJ), characterized by three helices emanating from a central junction. The right panel shows a triple-helix element at the 3′ terminus, in which a third RNA strand occupies the major groove and forms contiguous base-triplet interactions, contributing to structural stabilization of the RNA terminus. Nucleotide coloring is shown as follows: adenine in red, uracil in blue, cytosine in yellow, and guanine in green.

To assess potential functional features of the newly identified lncRNAs, we examined the tertiary structures of transcripts amenable to modeling. Among all candidates, TCONS_00371831 yielded a stable, biophysically plausible three-dimensional fold (Figure 8A). The model revealed an ordered architecture with long-range coaxial stacking, internal stem–loop elements, and well-defined tertiary contacts, consistent with structural motifs observed in known regulatory lncRNAs.

Closer structural inspection identified two conserved tertiary regulatory motifs (Figure 8B). First, a three-way junction (3WJ) adopting an archetypal parallel Y-shaped configuration, a hallmark frequently used by regulatory lncRNAs to scaffold protein complexes or mediate long-range structural communication. Second, a 3′ triple-helix element formed by base-triplet stacking, a motif associated with exonuclease resistance and transcript stabilization in other regulatory lncRNAs.

Together, these analyses define a set of novel prostate cancer-associated lncRNAs with coherent genomic, functional, and structural features. The presence of stable mRNA/miRNA interaction potential, combined with conserved tertiary motifs in representative transcripts, supports a model in which these lncRNAs may contribute to post-transcriptional regulation, immune modulation, and transcript stability in both circulating and tumor-resident contexts.

### 2.7. Unsupervised High-Dimensional Mapping Reveals Expanded Immunosuppressive Myeloid Populations in Prostate Cancer

**Figure 9:**
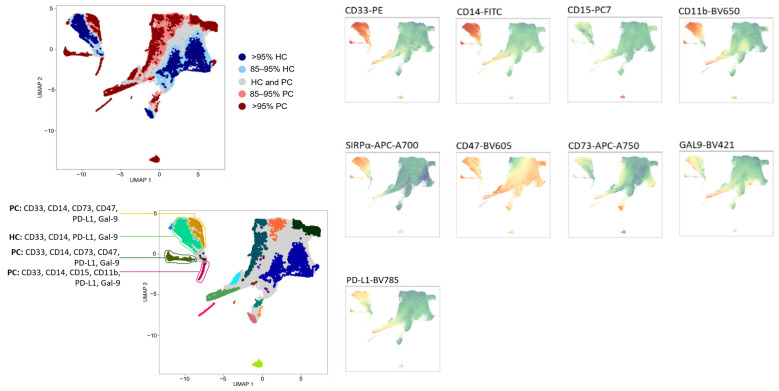
T-REX-guided UMAP analysis identifies prostate cancer-associated immunosuppressive myeloid subsets in peripheral blood. Peripheral blood mononuclear cells (PBMCs) from prostate cancer patients (PC, n = 12) and healthy controls (HC, n = 15) were analyzed using the T-REX plugin in FlowJo, which combines unsupervised UMAP dimensionality reduction with differential region enrichment based on multiparameter flow-cytometry data (CD33, CD14, CD15, CD11b, SIRPα, CD47, CD73, Galectin-9, and PD-L1). The top-left panel shows the sample origin projected onto the UMAP space, with regions enriched by >95% in PC (red) or HC (blue) regions, as identified by T-REX. Heatmaps on the right display marker expression intensity across the UMAP landscape, with green indicating low expression and red indicating high expression. Manual annotation of clusters (bottom-left), based on marker-intensity profiles, revealed PC-enriched myeloid populations co-expressing CD33 with CD14 and/or CD15, CD11b, and multiple immunoregulatory molecules, including CD47, CD73, Galectin-9, and PD-L1. These cancer-associated clusters are consistent with MDSC-like immunosuppressive phenotypes, supporting systemic myeloid remodeling in prostate cancer. Gating strategy and FMO-defined thresholds are shown in Appendix C, Figure A2.

Given the strong myeloid-skewing transcriptional programs identified—marked by neutrophil-attracting chemokines, systemic myeloid activation, and PBMC-derived signatures consistent with MDSC expansion—we next examined whether these molecular alterations translated into distinct immune cell populations at the single-cell phenotypic level. An unsupervised analysis was performed on PBMCs from prostate cancer patients (PC) and healthy subjects without a history of malignancy (HC), using an FMO (fluorescence-minus-one)-controlled gating strategy, to identify cell populations that differ between groups. The T-REX-guided UMAP projection revealed distinct spatial regions enriched either in PC (red) or HC (blue), as well as shared areas (gray), reflecting group-specific immune heterogeneity. Marker-expression heatmaps across the UMAP surface showed differential abundance of several myeloid-associated molecules, including CD33, CD14, CD15, CD11b, SIRPα, CD47, CD73, Gal-9, and PD-L1 (Figure 9). Effect size in T-REX analysis is reflected in the proportion of cells within regions that show >95% group-specific enrichment.

Based on marker intensity patterns and spatial localization, several populations of interest were identified. Regions enriched in PC exhibited myeloid clusters co-expressing CD33 together with CD14 and/or CD15, accompanied by elevated levels of multiple immunosuppressive checkpoints—particularly CD47, CD73, Gal-9, SIRPα, and PD-L1. These PC-dominant clusters correspond to immunosuppressive myeloid subsets consistent with MDSC-like phenotypes and were not observed in HC-enriched regions.

Together, these findings demonstrate that unsupervised high-dimensional analysis identifies expanded, immunoregulatory myeloid populations in the circulation of PC.

### 2.8. Assessment of Total MDSCs and Their Subsets in PBMCs from Healthy Individuals and Prostate Cancer Patients

**Figure 10:**
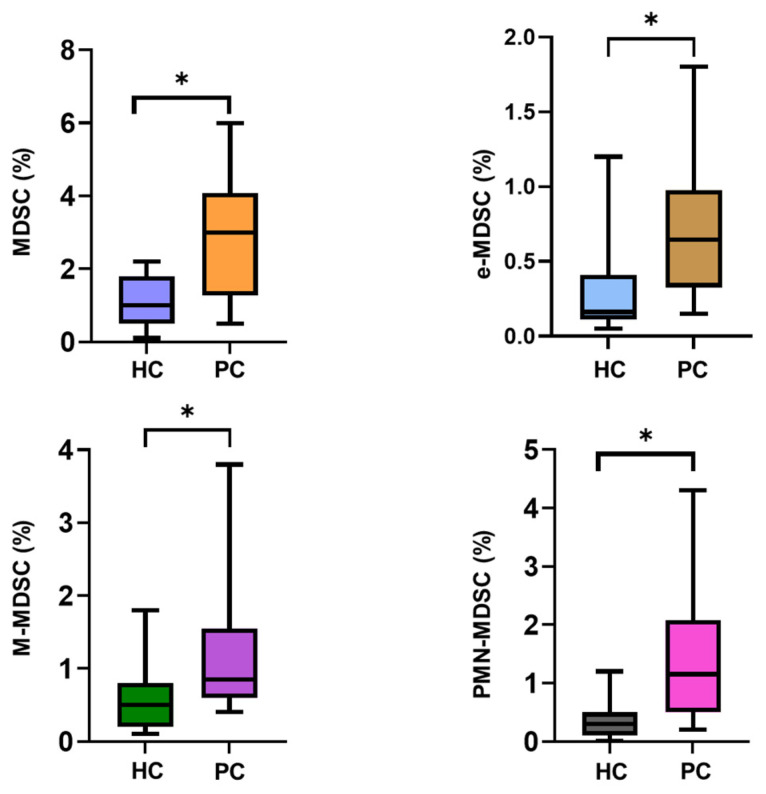
Quantitative assessment of circulating myeloid-derived suppressor cell (MDSC) subsets in prostate cancer. Percentages of total MDSCs and major subsets—early-stage MDSCs (e-MDSCs), monocytic MDSCs (M-MDSCs), and polymorphonuclear MDSCs (PMN-MDSCs)—were quantified in peripheral blood mononuclear cells (PBMCs) from healthy controls (HC, n = 15) and prostate cancer patients (PC, n = 12) using a standardized manual gating strategy. All MDSC subsets showed significantly higher frequencies in PC than in HC, with the greatest increase observed in PMN-MDSCs. Statistical significance was assessed using unpaired group comparisons, with an asterisk (*) denoting *p* < 0.05.

To validate the unsupervised T-REX/UMAP findings and determine whether these clusters corresponded to MDSC populations, a manual gating strategy was used to quantify total MDSCs and their major subsets in PBMCs from HC and PC. This targeted immunophenotyping analysis confirmed a significant systemic expansion of the MDSC compartment in PC (Figure 10). Total MDSCs were markedly increased in PC compared with HC (*p* < 0.05). Among the subsets, polymorphonuclear MDSCs (PMN-MDSCs) showed the greatest increase in PC in comparison with the HC group (*p* < 0.05), representing the dominant component driving overall expansion. Early-stage MDSCs (e-MDSCs) were also significantly higher in PC compared with HC (*p* < 0.05), and a similar difference was observed in monocytic MDSCs (M-MDSCs) between the groups (*p* < 0.05).

### 2.9. UMAP-Based High-Dimensional Profiling of Non-Lymphoid Myeloid Subsets Reveals Cancer-Enriched Immunosuppressive Populations

**Figure 11:**
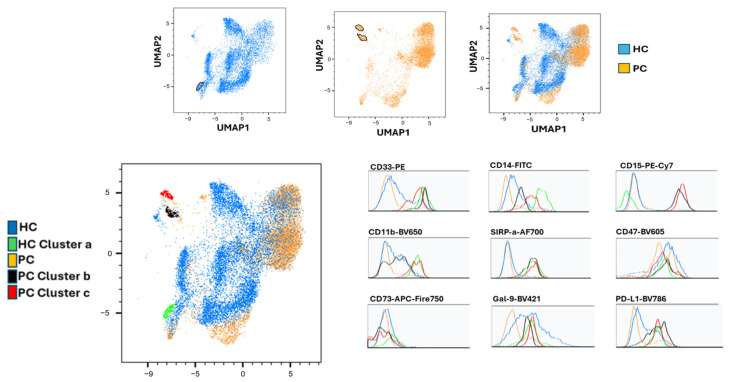
UMAP analysis of non-lymphoid, HLA-DR^−^ PBMCs identifies prostate cancer-associated myeloid clusters. UMAP dimensionality reduction was performed on peripheral blood mononuclear cells (PBMCs) after exclusion of lymphoid populations (CD3^+^, CD19^+^, CD20^+^, and CD56^+^) and antigen-presenting cells (HLA-DR^+^), thereby enriching for the mononuclear myeloid compartment. The resulting UMAP projection reveals discrete clusters differentially enriched in healthy controls (HC, n = 15) or prostate cancer patients (PC, n = 12). Two PC-enriched clusters (black and red) and one HC-enriched cluster (green) were annotated based on marker-expression profiles. Histogram overlays show increased expression of CD15, CD11b, CD47, PD-L1, CD73, and Galectin-9 within PC-enriched clusters, a phenotype consistent with immunosuppressive, MDSC-like myeloid states and supporting systemic myeloid remodeling in prostate cancer.

To refine the characterization of circulating myeloid populations, a more stringent analysis was performed by removing all lymphoid cells (CD3^+^, CD19^+^, CD20^+^, CD56^+^) and HLA-DR^+^ antigen-presenting cells. This filtration isolated the non-lymphoid, HLA-DR^−^ mononuclear fraction, enabling a focused examination of innate immune populations. UMAP dimensionality reduction in this compartment (Figure 11) revealed discrete clusters enriched either in PC or HC. The HC-enriched subset appeared as a single compact cluster (green), while two distinct cancer-associated clusters (black and red) emerged consistently across samples.

To further characterize these clusters, expression profiles of key myeloid and immunoregulatory markers were examined through histogram overlays. PC-specific clusters displayed increased expression of CD15 and CD11b, supporting a granulocytic/PMN-like identity, along with elevated CD47, PD-L1, CD73, and Gal-9—an inhibitory checkpoint repertoire highly consistent with immunosuppressive MDSC-like phenotypes. In contrast, the HC cluster showed lower baseline expression of these markers, aligning with a more homeostatic myeloid profile.

These findings indicate that, after excluding lymphoid and HLA-DR^+^ cells, the remaining myeloid compartment contains clearly segregated cancer-enriched subsets that exhibit the hallmark features of suppressive myeloid cells. The phenotypic overlap between these UMAP-derived clusters and the previously described immunosuppressive signatures in whole PBMC analysis reinforces the presence of distinct, functionally relevant myeloid alterations in prostate cancer, including a PC cluster with high expression of immunoregulatory receptors such as PD-L1.

### 2.10. Expression of Immunosuppressive Checkpoints Across Circulating MDSC Subsets

**Figure 12:**
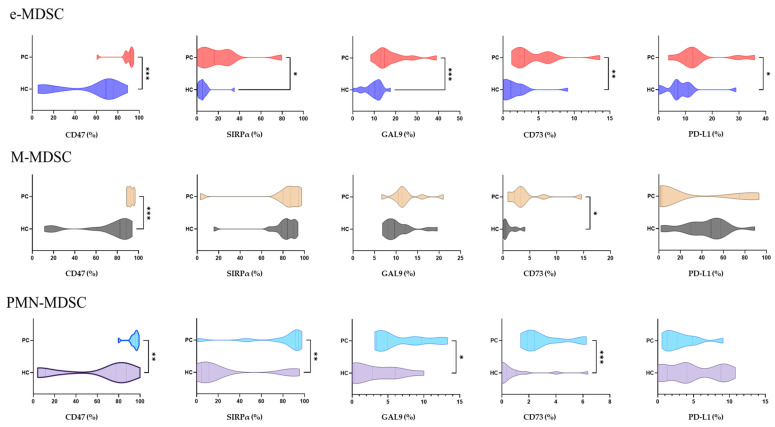
Violin plots depict the percentage of cells positive (%) for CD47, SIRPα, Galectin-9, CD73, and PD-L1 in early-stage myeloid-derived suppressor cells (e-MDSCs), monocytic MDSCs (M-MDSCs), and polymorphonuclear MDSCs (PMN-MDSCs) within peripheral blood mononuclear cells (PBMCs) from healthy controls (HC, n = 15) and prostate cancer patients (PC, n = 12). Across all three MDSC subsets, PC samples exhibit significantly higher frequencies of immunoregulatory marker-positive cells than HC samples. This expression pattern is consistent with enhanced immunosuppressive phenotypes and supports systemic remodeling of the circulating myeloid compartment in prostate cancer. Statistical significance is indicated as follows: * *p* < 0.05, ** *p* < 0.01, and *** *p* < 0.001.

Furthermore, we examined in detail whether circulating MDSC subsets differ in their expression of inhibitory receptors in PC compared with HC. We quantified the percentage of e-MDSCs, M-MDSCs, and PMN-MDSCs expressing CD47, SIRPα, Galectin-9, CD73, and PD-L1 in PBMCs from both groups (Figure 12). Across all three MDSC subsets, PC samples displayed elevated expression of multiple immunosuppressive molecules.

Among e-MDSCs, PC showed significantly higher expression of CD47, SIRPα, Galectin-9, CD73, and PD-L1 compared with HC (*p* < 0.05). M-MDSCs exhibited a more selective pattern, with increased expression of CD47 and CD73 in prostate cancer (*p* < 0.05), whereas Galectin-9, SIRPα, and PD-L1 showed no differences between groups. PMN-MDSCs also demonstrated marked upregulation of CD47, SIRPα, Galectin-9, and CD73 in PC samples compared with HC (*p* < 0.05). Overall, these results indicate that circulating MDSCs express higher levels of multiple inhibitory checkpoints in PC, supporting the presence of a systemically expanded and functionally suppressive myeloid compartment.

## 3. Discussion

Prostate cancer orchestrates a complex immunoregulatory landscape in which systemic inflammation coexists with a profoundly immune-excluded tumor microenvironment. Throughout this study, transcriptomic analyses are interpreted as context-defining and hypothesis-generating, whereas conclusions regarding myeloid expansion and immunosuppressive phenotypes are primarily supported by cytometric and single-cell phenotypic data. By integrating bulk transcriptomics from three independent cohorts, high-dimensional flow cytometry, unsupervised single-cell mapping, and machine-learning–based lncRNA discovery, our study provides a multi-layered view of how circulating myeloid populations, tumor-intrinsic transcriptional programs, and non-coding RNA circuits converge to sustain immune evasion. Together, these findings delineate a model in which systemic myeloid activation, checkpoint-rich suppressor expansion, and lncRNA-mediated regulatory architecture reinforce prostate cancer’s resistance to immune surveillance [28].

Importantly, the lncRNA-related findings presented in this study are derived from integrative computational analyses and should be interpreted as associative and hypothesis-generating, rather than as evidence of direct mechanistic causality. While predicted lncRNA–mRNA and lncRNA–miRNA interactions, as well as tertiary structure modeling, support the biological plausibility of regulatory roles for these transcripts, experimental validation will be required to determine whether and how specific lncRNAs functionally modulate immune signaling, myeloid suppressor biology, or relapse-associated transcriptional programs in prostate cancer.

Across PBMCs, tumor tissue, and relapse specimens, differential gene expression revealed a striking systemic-local dichotomy. In PBMCs, prostate cancer patients displayed robust upregulation of *IL1A*/*B*, *IL6*, *TNF*, *CXCL1*/*2*/*3*/*8*, *CCL2*/*3*/*20*, *VEGFA*, *PTGS2*, and *ARG1*, an inflammatory program characteristic of activated neutrophil-monocyte networks and early innate immune signaling [29]. Such peripheral inflammation is consistent with prior data indicating systemic myeloid priming in tumor-bearing hosts and is frequently associated with MDSC expansion and impaired T-cell priming [30]. In contrast, primary tumor tissue exhibited broad suppression of the inflammatory and chemokine axes, including downregulation of *CXCL2*/*6* and innate sensors such as *S100A8*/*P*, *MARCO*, and *PTGS1*, as well as loss of epithelial defense modules. This profile reinforces the long-recognized immune-excluded and chemokine-poor nature of prostate tumors, characterized by minimal effector-cell infiltration and substantial stromal exclusion despite systemic immune perturbation [28].

The relapse cohort yielded a markedly restricted, focused transcriptional signal when a stringent multiple-testing correction was applied. Notably, *CDH2* was the only transcript that retained statistical significance after FDR adjustment, underscoring its role as the most robust and reproducible molecular feature associated with biochemical recurrence in this dataset. Given its established function as a central mediator of epithelial–mesenchymal transition, CDH2 provides a biologically coherent link between relapse and the acquisition of invasive and plastic tumor states. In contrast, although several additional genes—including metabolic regulators and long non-coding RNAs—exhibited consistent nominal expression shifts, these signals did not survive FDR correction. They should therefore be interpreted as exploratory rather than definitive. Taken together, these findings suggest that biochemical recurrence is driven by a highly constrained transcriptional reprogramming dominated by *CDH2* upregulation. At the same time, broader alterations involving metabolic and non-coding RNA-related processes may reflect secondary or context-dependent changes that warrant independent validation. [31,32,33,34].

Pathway enrichment analysis confirmed that the PBMC inflammatory profile is not an isolated collection of dysregulated genes but a coherent signaling architecture dominated by IL-17, TNF, NF-κB, FoxO, and atherosclerosis-associated inflammatory pathways. GO terms further emphasized chemotaxis, stress-response regulation, and endothelial activation. The repeated involvement of JUN, FOS/FOSL1, ERG1/3, VEGFA, and CXCL family chemokines across multiple pathways demonstrates that prostate cancer induces a multi-axis inflammatory program in circulation [35]. This contrasts sharply with the immune-open dynamics seen in highly inflamed tumors such as melanoma and lung cancer and likely contributes to the limited efficacy of monotherapy immune checkpoint inhibition in prostate cancer [36].

Immune deconvolution of PBMC RNA revealed a prominent neutrophil-like signature exclusively in the cancer cohort, despite the physical removal of granulocytes during density-gradient isolation. This paradox is well described in cancer and typically reflects the presence of low-density neutrophils, which are known to represent PMN-MDSCs rather than mature neutrophils [37]. Consequently, our cytometry and T-REX/UMAP analyses identified CD33^+^, CD11b^+^, and CD14^+^/CD15^+^ myeloid clusters enriched in cancer patients and expressing suppressive molecules, including CD47, CD73, Gal-9, and PD-L1. Together, these data strongly support the interpretation that the “neutrophil” signal detected by deconvolution could correspond to circulating PMN-MDSCs, highlighting the tight integration between transcriptomic signatures and phenotypic suppressor expansion [37,38].

A recent work has further refined the understanding of polymorphonuclear myeloid-derived suppressor cells as highly plastic, systemically expanded populations that bridge chronic inflammation and immune suppression in cancer [39]. These studies demonstrate that PMN-MDSCs retain transcriptional and functional features distinct from mature neutrophils, enabling them to persist in circulation, infiltrate peripheral compartments, and exert potent inhibitory effects on T-cell activation. These findings align closely with our observation of neutrophil-like transcriptomic signatures in PBMCs and the cytometry-based expansion of checkpoint-rich PMN-MDSC populations, reinforcing the interpretation that circulating myeloid suppressors represent a key axis of systemic immune dysregulation in prostate cancer.

Weighted gene co-expression network analysis further underscored that these systemic and tumor-local programs are modular and coordinated. In PBMCs, a cancer-associated module enriched for inflammatory mediators and stress-response regulators (e.g., *CXCL2*, *PTGS2*, *PPP1R15A*) dominated the network architecture, while a negatively associated module comprising metabolic and protein homeostasis genes was suppressed. Tumor tissue networks were characterized by loss of structural, adhesion, and mechanotransduction modules—processes typically associated with epithelial integrity. Relapse-associated modules centered on chromatin remodeling (*BPTF*), DNA-repair regulators, and vesicular trafficking components, whereas non-recurrent tumors preserved mitochondrial, translational, and DNA-damage-response networks. These network-level findings imply that biochemical recurrence is driven not only by gain of pro-survival pathways but also by collapse of core structural and homeostatic programs [40,41].

A central contribution of this study is the identification and characterization of novel prostate cancer-associated lncRNAs with high-confidence regulatory potential expressed in both PBMCs and tumor tissues. Using a stringent pipeline integrating de novo transcript reconstruction, LncDC-based non-coding probability prediction, genomic-context annotation, and genome-wide target prediction via LncRTPred, IntaRNA, and scanMiR, we identified lncRNAs with high-confidence regulatory potential. Prior work has shown that lncRNAs modulate myeloid differentiation, MDSC accumulation, cytokine signaling, and immune checkpoint pathways [42,43,44,45]. In prostate cancer specifically, lncRNAs, including PCATs and AR-associated non-coding transcripts, contribute to disease progression and therapeutic resistance [46,47]. Our findings extend this framework by identifying lncRNAs expressed both in PBMCs and tumor tissue that harbor interaction profiles enriched for immune- and cancer-relevant genes.

Recent literature has further strengthened the view that long non-coding RNAs constitute active regulatory layers in cancer-associated immune remodeling rather than transcriptional bystanders. Contemporary studies have shown that lncRNAs participate in controlling inflammatory signaling, myeloid lineage commitment, and immunosuppressive phenotypes through context-specific expression and interaction networks, particularly under chronic cytokine stimulation [48,49]. These findings support the interpretation that the lncRNAs identified here, while computationally derived and hypothesis-generating, may represent components of broader regulatory architectures linking systemic inflammation, myeloid suppression, and prostate cancer progression.

Structural modeling of TCONS_00371831 provides additional mechanistic context for these observations. The predicted presence of a three-way junction—a classical RNA architectural motif—together with a putative stabilizing 3′ triple helix is consistent with features commonly observed in regulatory lncRNAs. However, experimental validation will be required to confirm these properties. Triple helices, as exemplified by MALAT1 and MENβ, have been shown to protect transcripts from exonucleolytic degradation, mediate long-range regulatory interactions, and, in some cases, substitute for poly(A) tails [50,51]. In this context, our observation that prostate cancer-associated lncRNAs include transcripts with such predicted structural features raises the possibility that lncRNAs may contribute to the persistence of inflammatory programs or influence MDSC maintenance. Notably, recent studies have reported that inflammatory cytokines, such as IL-6, can induce lncRNAs that promote MDSC expansion and suppressive function [43,44,52], suggesting a plausible mechanistic link between our transcriptomic findings and the observed immunophenotypic alterations.

Future experimental validation could include targeted perturbation of candidate lncRNAs under inflammatory conditions, assessment of RNA–protein interactions using immunoprecipitation-based approaches (such as RNA immunoprecipitation or crosslinking-assisted assays), and in-cell RNA structural probing methods to evaluate the formation and functional relevance of predicted tertiary motifs, including three-way junctions and triple-helix elements.

Unsupervised UMAP analyses revealed discrete prostate cancer-associated myeloid clusters, both in total PBMCs and within the non-lymphoid HLA-DR^−^ fraction. These cancer-enriched populations expressed CD15, CD11b, CD33, CD47, CD73, Gal-9, and PD-L1—precisely the profile expected of suppressive MDSCs [53,54]. Manual gating confirmed significant expansion of all three MDSC subsets, with PMN-MDSCs and e-MDSCs representing the dominant contributors. Notably, inhibitory checkpoint profiling demonstrated that e-MDSCs possess the broadest repertoire of suppressive molecules—including CD47, SIRPα, Gal-9, CD73, and PD-L1—suggesting that they may be functionally “hyper-armed” suppressors. This finding contrasts with the traditional focus on PMN-MDSCs as the major suppressive subset and highlights the need to integrate e-MDSCs into mechanistic and therapeutic models [23,55].

Although exploratory, these observations carry several potential therapeutic implications. First, the co-existence of hyperinflammatory PBMCs and an immune-excluded tumor microenvironment suggests that systemic and local immune dysfunction are coupled, explaining the limited activity of monotherapies targeting PD-1/PD-L1 in prostate cancer [56,57]. Second, the broad checkpoint repertoire of circulating MDSCs—including CD47–SIRPα and CD73-adenosine pathways—supports combination strategies aimed at suppressor-cell neutralization [58,59]. Third, the enrichment of lncRNA-driven regulatory programs in relapse raises the possibility of RNA-targeted interventions, including antisense oligonucleotides or small-molecule inhibitors of triple-helix or junctional motifs [60]. Finally, the strong link between MDSC expansion and transcriptomic inflammation suggests that early intervention in myeloid regulatory axes may delay or prevent recurrence [61].

Our study has limitations, including a modest sample size of PBMCs, a cross-sectional design, and reliance on computational predictions of lncRNA interactions and structural motifs. Accordingly, PBMC transcriptomic analyses were interpreted as exploratory and hypothesis-generating, rather than as definitive evidence. Importantly, key conclusions regarding systemic myeloid activation and MDSC expansion are not based solely on PBMC RNA-seq data, but are supported by independent, orthogonal validation using multiparameter flow cytometry, unsupervised UMAP/T-REX mapping, and manual gating of MDSC subsets. Experimental validation of lncRNA targets and in-cell structural probing will be essential to establish direct mechanistic roles. Likewise, integrating spatial transcriptomics or multiplex imaging will help resolve how circulating suppressor populations interface with tumor-resident immune cells.

In conclusion, our findings demonstrate that prostate cancer establishes a multi-layered immune-evasion program integrating systemic myeloid activation, checkpoint-rich MDSCs expansion, tumor immune quiescence, and lncRNA-mediated reprogramming. This multi-layered framework provides a foundation for integrated therapeutic approaches targeting both peripheral suppressor populations and the tumor microenvironment.

## 4. Materials and Methods

### 4.1. Patients and Sample Collection

To evaluate circulating MDSC levels, blood samples were collected from 12 prostate cancer patients and 15 healthy subjects without a history of malignancy, all at the Centro Médico Nacional de Occidente of the Instituto Mexicano del Seguro Social. Peripheral blood was collected in EDTA tubes for flow cytometry, whereas heparinized samples were processed for RNA extraction. All procedures adhered to the principles of the Declaration of Helsinki. Written informed consent was obtained from all participants, and the study protocol was approved by the institutional review board (Institutional Approval Number: R-2023-785-025).

Of the 12 prostate cancer patients included in the flow cytometry analysis, 8 donors also contributed PBMC samples for RNA sequencing, whereas the remaining 4 donors were analyzed exclusively by flow cytometry. Thus, RNA-seq and flow cytometry analyses partially overlapped but were not fully paired at the individual level.

### 4.2. Antibodies

The flow cytometry panel consisted of 11 markers: Lineage Cocktail (CD3, CD19, CD20, CD56; 363601), CD33 (366608), HLA-DR (307628), CD14 (325604), CD15 (301924), CD11b (301336), PD-L1 (329736), CD73 (344036), GAL9 (348920), SIRPα (323816), and CD47 (323120). All antibodies were obtained from BioLegend (San Diego, CA, USA; Appendix B, Table A1).

### 4.3. PBMC Isolation

PBMCs were isolated from both EDTA and heparinized blood samples using density gradient centrifugation with Histopaque-1077 (Sigma-Aldrich, 10771, St Louis, MO, USA). Whole blood was diluted 1:1 with sterile phosphate-buffered saline (PBS; pH 7.4) and gently layered onto Histopaque-1077 (Sigma-Aldrich, 10771, St Louis, MO, USA) in 15 mL conical tubes. Samples were centrifuged at 1800 rpm for 30 min at 20 °C with the brake off. The PBMC interphase was collected, transferred to a new tube, and washed twice with 5 mL PBS by centrifugation at 1800 rpm for 7 min at 20 °C. PBMCs isolated from EDTA-collected blood were processed immediately for flow cytometry (see Section 4.2 and Section 4.8). PBMCs isolated from heparinized blood were pelleted, resuspended in freezing medium (90% fetal bovine serum and 10% dimethyl sulfoxide), placed in a Mr. Frosty (ThermoScientific, 5100-0001, Waltham, MA, USA) isopropanol-based freezing container for controlled cooling (−1 °C/min) to −80 °C, and subsequently transferred to a liquid nitrogen storage tank (−196 °C) until RNA extraction.

### 4.4. RNA Extraction and Sequencing

Total RNA was isolated from 10 million PBMCs using the Quick-RNA Miniprep Kit (Zymo Research, R1055, Orange, CA, USA). RNA concentration and purity were assessed on a Synergy HT BioTek microplate reader (Winooski, VT, USA). Fourteen RNA samples were preserved in GenTegra RNAssure tubes (Pleasanton, CA, USA) and shipped to Novogene for library preparation and sequencing.

Poly(A)-selected libraries were prepared and sequenced on an Illumina NovaSeq X Plus (Hayward, CA, USA) platform to generate 150 bp paired-end reads. Sequencing depth ranged from 43 to 87 million reads per sample, corresponding to 6.5–13.1 Gb of raw data. All libraries passed Novogene’s internal QC filters, with Q30 scores above 92%.

### 4.5. Data Sources and Cohorts

Raw RNA-seq data were obtained from these three independent cohorts. The first was PBMC; our cohort consisted of samples collected for this study (8 prostate cancer patients and 6 healthy controls). The second was the prostate tissue transcriptomes from GEO (GSE22260), comprising 20 prostate cancer and 10 healthy prostate samples. The third was the relapse cohort (GSE120741), originally included 91 prostate cancer tissue samples, corresponding to 48 patients who developed biochemical recurrence within ~5 years of diagnosis and 43 patients without recurrence for ~10 years. One sample did not meet quality-control criteria, yielding a final set of 90 samples for analysis. A detailed summary of demographic, clinicopathological, and follow-up characteristics for all cohorts is provided in Appendix D, Table A2.

### 4.6. Transcriptomic Processing, Differential Expression, and System-Level Analyses

All transcriptomic analyses were performed in R (v4.4.1) using RStudio (v2025.09.1+401). For each cohort (PBMCs, prostate tissue, and relapse) independently, raw RNA-seq data were aligned to the GENCODE v48 GRCh38 reference genome and summarized at the gene level using Rsubread/featureCounts v2.22.1 [62], generating cohort-specific count matrices. Genes with very low counts were filtered out, and outlier samples were removed based on hierarchical clustering and principal component analysis (PCA). Count data were then normalized using the median-of-ratios method implemented in DESeq2 v1.48.1 [63]. Quality-control steps included dispersion estimation, Cook’s distance evaluation, MA plots, variance-stabilizing transformations (VST and rlog), PCA, and sample-to-sample distance heatmaps.

For all transcriptomic cohorts analyzed in this study (PBMCs, primary prostate tissue, and relapse tumors), standard quality-control procedures were applied independently before co-expression network inference. These procedures included hierarchical clustering and principal component analysis (PCA) of variance-stabilized expression data. They were used to identify and exclude samples with discordant global expression patterns before WGCNA.

Following quality-control filtering, co-expression networks were constructed using the final cohort-specific sample sets. Sample exclusions were performed prior to network construction using identical objective criteria across cohorts and are fully documented in the publicly available analysis scripts.

For the relapse cohort (GSE120741), differential expression analyses were performed using the full quality-controlled dataset (47 recurrent and 43 non-recurrent tumors; n = 90). Before WGCNA, additional sample-level outlier detection based on hierarchical clustering and principal component analysis identified four samples with discordant expression profiles (three recurrent and one non-recurrent tumors), which were excluded to improve network stability. Consequently, WGCNA in the relapse cohort was conducted on a final set of 86 samples (44 recurrent and 42 non-recurrent tumors), as reported in Figure 6.

Differential gene expression was performed separately for each cohort using DESeq2 with cohort-specific design formulas. For the PBMC cohort, the differential expression model was specified as design = ~ condition, with no additional covariates included due to limited sample size and incomplete per-sample clinical metadata beyond age. Log fold changes were refined using shrinkage estimators (apelgm [64], ashr [65]), and results were annotated to Ensembl gene symbols and descriptions via biomaRt v2.64.0 [66]. Gene co-expression networks were constructed within each cohort using the WGCNA framework [67], including soft-threshold selection, topological overlap calculation, module detection, module-trait correlation analyses, and export of module-specific networks for visualization in Cytoscape (v3.10.4) [68]. To assess the robustness of PBMC co-expression modules given the limited sample size, a leave-one-out (LOO) jackknife stability analysis was performed. The weighted gene co-expression network was reconstructed iteratively, each time removing one sample and re-estimating modules using identical parameters (signed network, fixed soft-threshold power, and merge cut height). Module stability was evaluated at two levels: (i) preservation of module eigengene correlations with disease status across LOO iterations, and (ii) preservation of module gene composition using Jaccard similarity relative to the full network. These analyses were used to identify stable modules suitable for biological interpretation.

Pathway enrichment and immune deconvolution were performed independently for each cohort to resolve systems-level signaling architecture and circulating immune shifts. Enrichment analyses were conducted with PathfindR v2.6.0 [69] using KEGG and Go-All gene sets, leveraging the KEGG interaction network for active-subnetwork identification; gene–pathway associations were visualized through circular network layouts integrating pathway significance with gene-level log2 fold changes. For deconvolution, TPM-normalized matrices were analyzed with xCell2 v1.0.11 [70] and LM22 matrix [71] to obtain enrichment scores across 22 immune subsets, retaining only cell types with >90% reference gene overlap and evaluating group differences using Welch’s *t*-tests with Benjamini–Hochberg correction. Although all procedures were applied uniformly across PBMCs, prostate tissue, and relapse cohorts, only the PBMC dataset yielded statistically significant pathway-level activation and immune-cell perturbations. It thus served as the only cohort subjected to detailed systems-level interpretation.

To avoid batch effects arising from differences in tissue type, sequencing origin, and experimental protocols, all transcriptomic analyses were conducted using three fully independent pipelines for PBMCs, primary prostate tissue, and relapse tumors. Each cohort was processed separately from raw read alignment through normalization, differential expression, network analysis, pathway enrichment, and immune deconvolution. No cross-cohort normalization, batch correction, or joint differential modeling was performed, as such approaches would be inappropriate across biologically distinct compartments (blood versus tumor tissue) and could mask genuine biological differences.

Within the PBMC cohort, all samples were collected during the same calendar year (2024), processed using identical protocols, and sequenced together in a single Novogene submission, resulting in no internal batch or run-level stratification. Accordingly, batch-correction methods were not applied. Principal component analysis and hierarchical clustering of variance-stabilized and rlog-transformed expression data were used exclusively as quality-control steps to assess internal structure, detect outliers, and evaluate potential hidden technical effects; these analyses did not reveal clustering driven by technical artifacts (Appendix E, Figure A3).

Context-specific transcriptional programs were therefore inferred by comparison of independently derived results across cohorts, rather than through direct statistical integration or cross-cohort contrasts.

### 4.7. Discovery and Functional Characterization of Long Non-Coding RNAs (lncRNAs)

Putative lncRNA transcripts were obtained through a streamlined preprocessing and assembly workflow adapted from the LncDC pipeline [72]. Quality control and read preprocessing were performed with Trim Galore v0.6.5 [73], which integrates Cutadapt v3.5 [74] for adaptor removal and quality trimming, and FastQC v0.12.1 [75] for reporting. After trimming, only high-quality paired reads were retained for downstream alignment and transcriptome reconstruction. Reads were aligned to the GRCh38 reference genome (GENCODE v49) using both STAR v2.7.5a [76] and HISAT2 v2.2.1 [77], and assembled with Cufflinks v2.2.1 [78] and Stringtie v2.1.2 [79]. Consensus transcripts shared between both assembly routes were retained, excluding those shorter than 200 nt, single-exon transcripts, or transcripts detected in normal tissues. Candidate sequences were extracted with gffread v.0.12.7 [80] and filtered against NONCODEv6 to remove previously annotated lncRNAs.

Transcripts were classified as coding or non-coding using LncDC. This machine learning model integrates sequence features, secondary structure descriptors, and translated-protein attributes to achieve state-of-the-art lncRNA prediction accuracy (~99%).

Predicted lncRNAs were mapped to GRCh38 using minimap2 v2.28 [81], and genomic context was defined (antisense, sense-overlapping, sense-intronic, bidirectional, intergenic) through strand-aware overlap analysis with bedtools v2.31.1 [82]. Functional inference was performed by combining LncRTPred [83] (lncRNA-mRNA interaction potential), IntaRNA v3.4.1 [84] (minimum hybridization energy), and scanMiR v1.16.0 [85] (miRNA-binding prediction), generating an integrated map of putative RNA-RNA interactions relevant to cancer and immune regulation.

To complement these functional predictions, we additionally sought to explore the structural properties of selected candidates, as three-dimensional conformation can provide mechanistic insight into RNA-RNA recognition, stability, and regulatory potential. For this purpose, we employed RhoFold+ [86], one of the most advanced deep-learning frameworks currently available for RNA tertiary-structure prediction. Because the method has been benchmarked and validated primarily on RNA molecules ranging from ~16 to 300 nucleotides, only the shortest high-confidence lncRNA in our dataset met the compatibility requirements for accurate modeling. This sequence was submitted to the RhoFold+ online server, yielding a stable and well-resolved three-dimensional RNA model suitable for downstream structural interpretation.

### 4.8. Flow Cytometry

Two million PBMCs isolated from EDTA-collected blood were stained for flow cytometry analysis. To minimize nonspecific interactions, cells were incubated for 5 min at 20 °C with 5 µL True-Stain Monocyte Blocker (BioLegend, 426102, San Diego, CA, USA) to suppress dye-associated binding on monocytes and 5 µL Human TruStain FcX (BioLegend, 422302, San Diego, CA, USA) to block FcγR-mediated IgG binding. Without washing, the cell suspension was mixed directly with the antibody staining cocktail containing the full 11-marker panel (see Section 4.2) and incubated for 20 min at 20 °C in the dark. After staining, cells were washed with 2 mL PBS and centrifuged at 1800 rpm for 7 min at 20 °C. Then, the cells were resuspended in 250 µL PBS (Sigma-Aldrich, P3813-5X10PAK, Darmstadt, Germany) and immediately acquired on a CytoFLEX flow cytometer (Beckman Coulter Cytometer, Miami, FL, USA).

**Figure A2:**
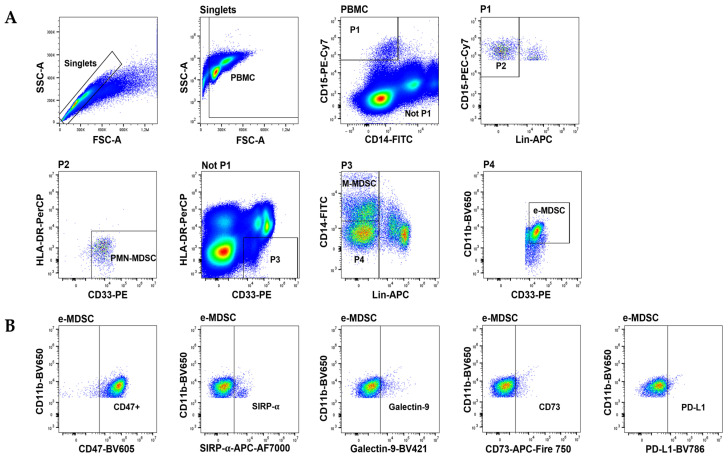
Flow cytometric identification and phenotypic characterization of human circulating MDSC subsets in prostate cancer. (**A**) Sequential gating workflow applied to PBMC samples. Singlets were first isolated by FSC-A x SSC-A discrimination, followed by selection of the PBMC compartment. Within PBMCs, lineage-negative (Lin^−^; CD3^−^ CD19^−^ CD20^−^ CD56^−^) myeloid cells were separated into CD15^+^/CD14^−^ (P1) and CD15^−^/CD14^+^/dim (not P1) fractions. From the CD15^+^/HLA-DR^−^/CD33^+^ compartment (P2), PMN-MDSCs were identified. The CD15^−^ fraction was further resolved into HLA-DR^−^/CD33^+^ M-MDSCs (P3) and the CD14^−^/Lin^−^/HLA-DR^−^/CD11b^+^ population corresponding to e-MDSCs (P4) [87]. (**B**) Expression of immunoregulatory checkpoints in e-MDSCs. Representative dot plots show surface staining for CD47, SIRPα, Galectin-9, CD73, and PD-L1, demonstrating differential expression patterns across the early-stage suppressor subset. Gates reflect the threshold of positive staining defined by matched FMO controls. Collectively, these data illustrate a harmonized, reproducible gating strategy that enables high-fidelity discrimination of PMN-MDSC, M-MDSC, and e-MDSC subsets and their associated inhibitory receptor profiles.

**Figure A3:**
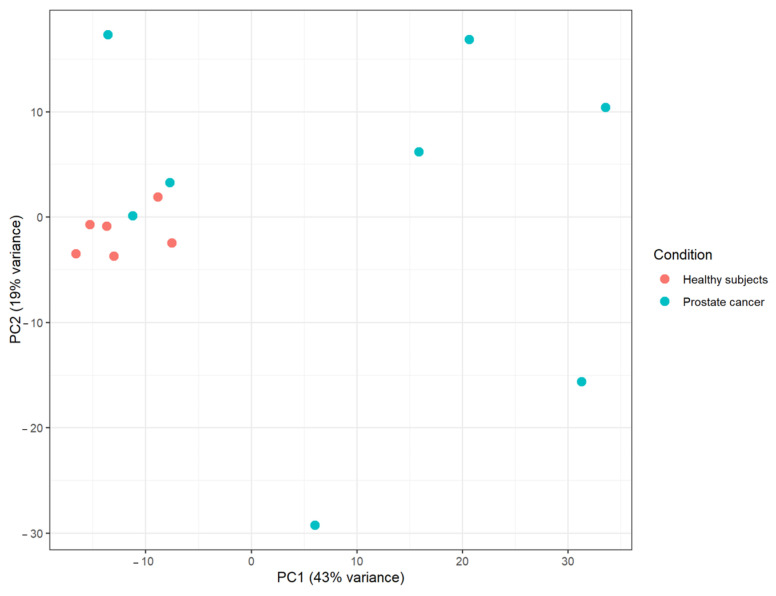
Principal component analysis of PBMC RNA-seq data. Principal component analysis (PCA) was performed on variance-stabilized (VST) gene expression values from peripheral blood mononuclear cell (PBMC) samples of prostate cancer patients (n = 8) and healthy subjects (n = 6). All samples were collected in the same calendar year (2024), processed using identical protocols, and sequenced together in a single Novogene submission. Principal components 1 and 2 account for 43% and 19% of the total variance, respectively, and show separation consistent with biological condition. Although a subset of prostate cancer samples exhibits increased dispersion along PC1 and PC2, quality-control analyses—including dispersion estimation, Cook’s distance evaluation, and hierarchical clustering—did not identify these samples as technical outliers. No clustering suggestive of batch or other technical artifacts was observed.

**Figure A4:**
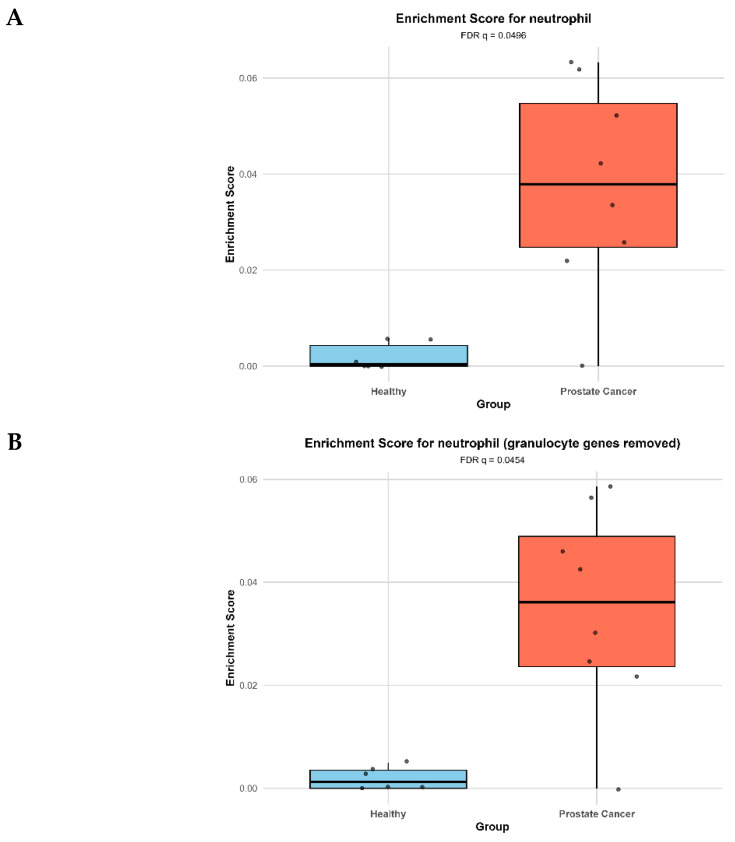
Robustness analysis of neutrophil enrichment in PBMCs. Neutrophil enrichment scores estimated by xCell2 in PBMC samples from healthy controls and prostate cancer patients before (**A**) and after (**B**) removal of canonical granulocyte granule and lineage genes. Despite the exclusion of 25 well-established granulocyte-associated genes, the neutrophil-like signal remains significantly elevated in prostate cancer PBMCs (FDR q = 0.045), indicating that a small subset of contamination markers does not drive the enrichment.

Flow cytometry data were analyzed using FlowJo v10.10 (Becton, Dickinson & Company, Ashland, OR, USA) using a standardized, widely adopted gating strategy for human MDSC identification (Appendix C, Figure A2) [87]. Fluorescence-minus-one (FMO) controls were used to define positivity thresholds and mean fluorescence intensity (MFI) for all markers.

High-dimensional cytometry analysis was performed on the PBMC population using concatenated samples in FlowJo. Initial quality control was conducted using PeacoQC (Peak-based selection of high-quality cytometry data) [88], followed by sequential gating on single events and selection of the PBMC compartment. Dimensionality reduction was performed using the built-in UMAP (Uniform Manifold Approximation and Projection) implementation in FlowJo [89]. UMAP analyses were conducted using the default parameters provided by FlowJo, without manual tuning of neighborhood size, distance metric, minimum distance, or random seed. This approach was chosen to ensure reproducibility and maintain consistency with widely used workflows for high-dimensional flow cytometry data.

Unsupervised population structure was further explored using Phenograph clustering, and the resulting clusters were evaluated using the Cluster Explorer tool [90]. Differential population enrichment between prostate cancer patients and healthy controls was assessed using the T-REX plugin [91], which identifies regions of maximal group-specific enrichment rather than enforcing hard cluster boundaries. Cluster robustness was evaluated based on reproducibility of enriched regions across repeated analyses using identical preprocessing and parameters, as well as concordance with independently validated populations obtained by manual gating and quantitative statistical analyses.

### 4.9. Statistical Analysis

All statistical analyses were performed in R (v4.4.1) unless otherwise stated. For flow cytometry, group comparisons between prostate cancer patients and healthy controls were performed using Mann–Whitney U tests or Welch’s t-tests when variance heterogeneity was present. Positivity thresholds were defined using fluorescence-minus-one (FMO) controls, and mean fluorescence intensity (MFI) differences were evaluated using the same statistical framework. Differences between groups were assessed using the corresponding statistical test, and values < 0.05 were considered statistically significant. All statistical analyses of flow cytometry assays were performed using GraphPad Prism (v10.3.1; LLC d.b.a. Dotmatics, San Diego, CA, USA).

For RNA-seq data, DESeq2’s dispersion modeling and Wald tests were used to compute differential expression, with multiple testing controlled by the Benjamini–Hochberg (BH) method (FDR < 0.05). Additional details supporting the differential expression results are provided in Supplementary Materials Tables S1–S3. Module–trait associations in WGCNA were evaluated using Pearson correlations with Student-transformed *p*-values (<0.05). Immune deconvolution scores generated by xCell2 were compared between groups using Welch’s *t*-tests with BH correction across all cell subsets.

For lncRNA analyses, LncDC, LncRTPred, and IntaRNA generate predictive probabilities, interaction likelihoods, or thermodynamic energies without associated inferential statistics and were therefore used descriptively. In contrast, scanMiR performs formal statistical testing: site-level empirical *p*-values were obtained from the distribution of −logKd values and corrected using Independent Hypothesis Weighting (IHW; FDR < 0.05), and miRNA–lncRNA pair-level significance was assessed by aggregating site-level *p*-values with the Aggregated Cauchy method, followed by Benjamini–Hochberg correction (q < 0.05). Only significant sites and pairs were retained for downstream analyses. Across all analyses, *p* < 0.05 or FDR < 0.05 was considered statistically significant.

## Figures and Tables

**Table 1:** Functional characterization of predicted lncRNAs ^1^.

Description	Length	Chrom	Start	End	Strand	Class	Nearest Gene	Ensembl_ID	IntaRNA Targets	Energy (kcal/mol)
PBMCs										
TCONS_00371831	216	2	237,785,441	237,785,747	+	intergenic			LBR,ITGB1,CAB39	(−29)(−18)(−13)
TCONS_00541561	249	6	70,570,816	70,576,857	+	intergenic			GALNT1,LBR,GNAI1	(−18)(−15)(−13)
TCONS_00125660	309	11	67,504,521	67,504,860	+	antisense	PITPNM1	ENSG00000110697	HABP4,PPP3CB,CD164	(−28)(−24)(−15)
TCONS_00372669	363	2	238,269,129	238,269,488	−	intergenic			RSK2,XPOT,GMCL1	(−28)(−22)(−15)
TCONS_00651951	421	Y	13,271,267	13,278,359	−	intergenic			ACSL3,ZMYND11,SNX14	(−22)(−22)(−19)
TCONS_00277876	460	17	2,949,179	2,949,764	−	antisense	RAP1GAP2	ENSG00000132359	CDC42,C9orf72,HERC4	(−26)(−16)(−15)
TCONS_00542118	475	6	43,636,844	43,639,182	−	antisense	MAD2L1BP	ENSG00000124688	RSK2,CNOT6,LBR	(−36)(−21)(−21)
TCONS_00629693	498	9	133,057,176	133,057,770	−	antisense	GTF3C5	ENSG00000148308	HERC4,CSNK2A1,SDE2	(−28)(−23)(−21)
TCONS_00372672	521	2	239,124,560	239,125,276	−	intergenic			CNBP,TMEM33,CALM1	(−19)(−18)(−17)
TCONS_00194633	603	14	88,607,460	88,608,089	−	antisense	ZC3H14	ENSG00000100722	RSK2,HERC4,FMR1	(−59)(−54)(−33)
TCONS_00063517	627	1	44,802,011	44,802,691	−	antisense	PLK3	ENSG00000173846	CSTF2T,FMR1,CEBPG	(−41)(−40)(−27)
TCONS_00629105	706	9	133,099,118	133,099,844	+	antisense		ENSG00000285245	CSTF2T,FMR1,GMCL1	(−46)(−36)(−29)
TCONS_00125288	765	11	14,885,967	14,888,699	+	antisense	CYP2R1	ENSG00000186104	CSTF2T,STT3B,LIG4	(−66)(−32)(−23)
TCONS_00372670	1281	2	239,093,715	239,094,741	+	antisense	HDAC4	ENSG00000068024	CSTF2T,FBN1,PPP6C	(−39)(−34)(−20)
TCONS_00395970	3647	21	41,407,990	41,420,087	−	antisense	MX2	ENSG00000183486	SIRT3,PCK2,CERS4	(−147)(−33)(−28)
Tissue										
TCONS_00248077	243	16	19,246,119	19,246,360	+	sense_intronic	SYT17	ENSG00000103528	RSK2,PPM1A,ZMYND11	(−172)(−20)(−14)
TCONS_00589880	267	7	6,691,215	6,691,482	+	antisense	ZNF12	ENSG00000164631	CSTF2T,ZMYND11,LBR	(−22)(−18)(−13)
TCONS_00162777	275	12	48,971,956	48,972,523	−	intergenic			SOD2,CEBPG,HERC4	(−23)(−16)(−16)
TCONS_00590288	302	7	150,999,718	151,000,074	+	sense_intronic	NOS3	ENSG00000164867	HERC4,ITGB1,FMR1	(−72)(−59)(−50)
TCONS_00590074	369	7	76,996,891	76,997,307	+	intergenic			BMI1,PPP3CB,CEBPG	(−113)(−99)(−67)
TCONS_00092197	400	10	132,364,305	132,364,741	−	antisense	LRRC27	ENSG00000148814	HERC4,XPO1,CMTM6	(−76)(−57)(−49)
TCONS_00065281	532	1	226,978,281	226,979,189	−	antisense		ENSG00000288674	FDX1,GNG2,ANGEL2	(−85)(−73)(−58)
TCONS_00398427	556	20	19,924,040	19,924,654	−	antisense	RIN2	ENSG00000132669	CNOT6,HERC4,FMR1	(−82)(−79)(−51)
TCONS_00137682	588	12	62,958,750	62,959,550	+	intergenic			CSTF2T,FMR1,RSK2	(−44)(−30)(−23)
TCONS_00407292	604	21	42,439,474	42,440,170	−	antisense	UBASH3A	ENSG00000160185	IL18R1,PLCB1,HERC4	(−76)(−68)(−63)
TCONS_00407190	763	21	41,475,060	41,475,926	+	antisense	TMPRSS2	ENSG00000184012	PAFAH1B2,HERC4,CSTF2T	(−112)(−101)(−79)
TCONS_00331002	792	19	47,513,215	47,519,562	+	antisense	NAPA	ENSG00000105402	CSTF2T,PAFAH1B2,HERC4	(−52)(−36)(−24)
TCONS_00065280	803	1	226,977,580	226,978,785	−	antisense		ENSG00000288674	PPP2R5E,PAFAH1B2,PHF6	(−105)(−86)(−64)
TCONS_00522040	1168	5	15,846,410	15,851,647	−	antisense	FBXL7	ENSG00000183580	LBR,CSTF2T,NUDT21	(−34)(−30)(−24)
TCONS_00381284	1471	2	238,901,950	238,903,658	+	sense_intronic	TWIST2	ENSG00000233608	RSK2,UBE2D3,PERP	(−75)(−70)(−69)
TCONS_00608643	1628	8	53,549,960	53,552,709	−	intergenic			CSTF2T,ZMYND11,ZBTB1	(−35)(−27)(−22)
TCONS_00161804	2092	12	132,155,565	132,158,161	−	intergenic			PERP,UBE2D3,FMR1	(−70)(−57)(−49)
TCONS_00555128	2231	6	170,397,903	170,400,405	+	sense_intronic	FAM120B	ENSG00000112584	RAP1A,CSTF2T,NUDT21	(−31)(−30)(−24)
TCONS_00590782	3299	7	158,336,126	158,341,789	−	sense_intronic	PTPRN2	ENSG00000155093	DDAH2,RNF215,PTK6	(−29)(−28)(−27)
TCONS_00554941	3684	6	42,887,581	42,889,932	+	sense_overlapping	RPL7L1	ENSG00000146223	PCGF2,ZFP36,GNB2	(−41)(−35)(−27)
TCONS_00129676	4161	12	7,115,273	7,119,124	−	sense_overlapping	RBP5	ENSG00000139194	NOX1,TRIM27,HPN	(−32)(−32)(−24)
TCONS_00161805	4663	12	132,155,565	132,243,321	−	sense_overlapping	GALNT9	ENSG00000182870	EREG,RB1,GNG12	(−65)(−58)(−49)
TCONS_00162601	9091	12	123,510,233	123,513,312	+	antisense	RILPL1	ENSG00000188026	COPA,GLDC,PIP4K2C	(−70)(−66)(−61)

^1^ High-confidence lncRNAs identified in the PBMC and tumor tissue cohorts using LncDC (non-coding probability > 0.99) are shown. For each transcript, genomic coordinates were annotated against the GRCh38 reference genome and classified by transcript length, chromosomal location, strand orientation, and genomic context (intergenic, antisense, sense-intronic, or sense-overlapping), along with the nearest protein-coding gene and its Ensembl identifier. Predicted lncRNA-RNA interactions were inferred using LncRTPred, and the three most cancer- or immune-relevant targets per lncRNA are reported. RNA–RNA interaction stability was further evaluated using IntaRNA, with minimal hybridization energies (kcal/mol) shown for each prioritized target pair. The relapse cohort was excluded from lncRNA discovery due to insufficient transcriptomic coverage.

## Data Availability

The RNA-seq data generated from PBMC samples in this study are not publicly available due to patient privacy restrictions, but are available from the corresponding author upon reasonable request. Publicly available datasets analyzed in this study were obtained from the Gene Expression Omnibus (GEO) under the accession numbers GSE22260 and GSE120741. All scripts used for data processing and analysis, as well as the sequences of the lncRNAs identified in this study, are available at https://github.com/Linfopeter/Prostate_cancer (accessed on 27 January 2026).

## Supplementary Materials

The following supporting information can be downloaded at: https://www.mdpi.com/article/10.3390/ijms27031511/s1.

## Abbreviations

The following abbreviations are used in this manuscript:

MDSC	Myeloid-derived suppressor cells
PMN-MDSC	Polymorphonuclear MDSC
e-MDSC	Early-stage MDSC
M-MDSC	Monocytic MDSC
FMO	Fluorescence minus one
MFI	Mean fluorescence intensity
DAMP	Damage-associated molecular pattern
EMT	Epithelial–mesenchymal transition
COX-2	Cyclooxygenase 2
lncRNAs	Long non-coding ribonucleic acids
3WJ	Three-way junction
HC	Healthy subjects (controls)
PC	Prostate cancer patients
ME	Module
GO	Gene Ontology
PBS	Phosphate-buffered solution
BH	Benjamini–Hochberg method
GEO	Gene Expression Omnibus

## Appendix B

**Table A1:** Reagents used for flow cytometry.

Specificity	Clone	Fluorochrome	Purpose	Cat	Dilution
CD3, CD19, CD20, CD56 (Lineage)	UCHT1, HIB19, 2H7, 5.1H11	APC	Lymphocyte exclusion	363601 (BioLegend)	1:16
CD33	P67.6	PE	Myeloid cells	366608 (BioLegend)	1:16
HLA-DR	L243	PerCP	Exclusion of APCs	307628 (BioLegend)	1:8
CD14	HCD14	FITC	Monocytes	325604 (BioLegend)	1:16
CD15	HI98	PE-Cy7	Granulocytes	301924 (BioLegend)	1:16
CD11b	ICRF44	BV650	e-MDSCs	301336 (BioLegend)	1:4
PD-L1	29E.2A3	BV785	Immunoregulation	329736 (BioLegend)	1:4
CD73	AD2	APCFire750	Immunoregulation	344036 (BioLegend)	1:4
Galectin-9	9M1-3	BV421	Immunoregulation	348920 (BioLegend)	1:2
SIRPα	SE5A5	Alexa Fluor700	Immunoregulation	323816 (BioLegend)	1:16
CD47	CC2C6	BV605	Immunoregulation	323120 (BioLegend)	1:32

Comprehensive antibody panel used for multiparametric flow-cytometric identification and phenotyping of circulating myeloid-derived suppressor cell (MDSC) subsets. The table summarizes each marker’s specificity, clone, fluorochrome conjugate, and biological purpose within the gating strategy. Lineage markers (CD3, CD19, CD20, and CD56) conjugated to APC were used to exclude lymphoid cells. Myeloid markers (CD33, CD14, CD15, CD11b, and HLA-DR) enabled discrimination between classical monocytes, granulocytic populations, and antigen-presenting cells. Immunoregulatory checkpoint molecules relevant to MDSC suppressive function, including PD-L1, CD73, Galectin-9, SIRPα, and CD47, were included to define functional states associated with immune evasion. For each antibody, the catalog number (BioLegend) and the final staining dilution used in the assay are provided.

## Appendix D

**Table A2:** Demographic and clinical characteristics of the three transcriptomic cohorts analyzed in this study.

**Cohort**	PBMC (this study)	Tissue (public)	Relapse (public)
**Data source**	This study	GSE22260	GSE120741
**Sample type**	PBMCs	Prostate tissue	Prostate tissue
**Original cohort size**	14	30	101
**Prostate cancer samples**	8	20	47
**Number of healthy controls**	6	10	43
**Age at diagnosis, years**	67.5 (51–71)	60 (39–73)	64 (47–73) *
**Gleason score distribution**	6–7	6–9	6–9 *
**Tumor stage (TNM)**	T1–T2, N0	T2–T3, N0–N1	T2–T4, N0 *
**Biochemical recurrence status**	NA	NA	PSA-defined *
**Time to recurrence (months)**	NA	NA	41 (14–74) *
**Follow-up duration (months)**	NA	NA	150 (70–191) *

Summary of available demographic and clinicopathological characteristics across the three transcriptomic cohorts. PBMC samples were generated and analyzed in the present study. Tissue and relapse cohorts correspond to publicly available prostate cancer datasets (GSE22260 and GSE120741, respectively). Values are reported as median (range) when applicable. Per-sample metadata beyond age and disease status were not uniformly available across all cohorts and are therefore summarized at the cohort level. Clinical and follow-up characteristics for the relapse cohort are reported as described in the original Porto cohort publication. The present study analyzed a subset of 90 relapse samples with available, quality-controlled transcriptomic data; patient-level clinical identifiers were not available to enable recalculating clinical summaries for this subset. NA indicates information not applicable or not available for the corresponding cohort. Asterisks (*) denote values derived from the original relapse cohort publication. Bold formatting is used solely to distinguish the descriptor column from cohort-specific data and does not imply analytical or biological emphasis.

## Appendix F

**Table A3:** Stability of PBMC Co-expression Module–Trait Associations Assessed by Leave-One-Out Jackknife Analysis.

Module	mean_cor	sd_cor	min_cor	max_cor	prop_same_sign_vs_full
MEturquoise	−0.77285	0.049878	−0.83329	−0.65944	1
MEblue	0.627098	0.086398	0.508458	0.743487	1
MEorange	0.194098	0.383003	−0.43644	0.898417	0.272727
MEskyblue3	0.079957	0.388913	−0.72051	0.516068	0.727273
MEgrey60	0.025156	0.493752	−0.72446	0.641504	0.636364

Leave-one-out (LOO) jackknife analysis of PBMC co-expression module–trait associations. For each LOO iteration, the weighted gene co-expression network was reconstructed after removing one sample, and module eigengene correlations with disease status (cancer vs. healthy) were recalculated using identical parameters. The table summarizes correlation estimates, variability across iterations, and preservation of correlation sign relative to the full network. Only modules showing consistent eigengene–trait associations across all iterations were considered robust and interpreted in the main analysis.

## Appendix G

**Table A4:** Leave-One-Out Jackknife Evaluation of PBMC Module Gene-Set Preservation Using the Jaccard Index.

ref_module	median_jaccard	min_jaccard	max_jaccard
turquoise	0.462885	0.252719	0.533985
blue	0.345064	0.26286	0.412484
grey60	0.132353	0.04902	0.250847
orange	0.128889	0.071713	0.198758
skyblue3	0.038889	0.010256	0.22807

Leave-one-out (LOO) assessment of PBMC co-expression module composition stability. Module preservation was evaluated by quantifying gene-set overlap between the reference network and each LOO-derived network using the Jaccard similarity index. For each reference module, the best-matching module in each LOO iteration was identified, and summary statistics of Jaccard overlap are reported. Higher Jaccard values indicate greater preservation of module gene composition across LOO iterations.
